# Altered microbial biogeography in an innate model of colitis

**DOI:** 10.1080/19490976.2022.2123677

**Published:** 2022-09-26

**Authors:** Antonia Boger-May, Theodore Reed, Diana LaTorre, Katelyn Ruley-Haase, Hunter Hoffman, Lauren English, Connor Roncagli, Anne-Marie Overstreet, David Boone

**Affiliations:** a Department of Microbiology and Immunology, Indiana University School of Medicine, South Bend, IN, USA; b Department of Biology, University of Notre Dame, South Bend, IN, USA

**Keywords:** A20, laser capture microdissection, TRAG, microbiome, innate immunity, biogeography, colitis, inflammatory bowel disease, IBD

## Abstract

Changes in the spatial organization, or biogeography, of colonic microbes have been observed in human inflammatory bowel disease (IBD) and mouse models of IBD. We have developed a mouse model of IBD that occurs spontaneously and consistently in the absence of adaptive immunity. Mice expressing tumor necrosis factor-induced protein 3 (TNFAIP3) in intestinal epithelial cells (villin-TNFAIP3) develop colitis when interbred with Recombination Activating 1-deficient mice (RAG1^−/−^). The colitis in villin-TNFAIP3 × RAG1^−/−^ (TRAG) mice is prevented by antibiotics, indicating a role for microbes in this innate colitis. We therefore explored the biogeography of microbes and responses to antibiotics in TRAG colitis. Laser capture microdissection and 16S rRNA sequencing revealed altered microbial populations across the transverse axis of the colon as the inner mucus layer of TRAG, but not RAG1^−/−^, mice was infiltrated by microbes, which included increased abundance of the classes Gammaproteobacteria and Actinobacteria. Along the longitudinal axis differences in the efficacy of antibiotics to prevent colitis were evident. Neomycin was most effective for prevention of inflammation in the cecum, while ampicillin was most effective in the proximal and distal colon. RAG1^−/−^, but not TRAG, mice exhibited a structured pattern of bacterial abundance with decreased Firmicutes and Proteobacteria but increased Bacteroidetes along the proximal to distal axis of the gut. TRAG mice exhibited increased relative abundance of potential pathobionts including *Bifidobacterium animalis* along the longitudinal axis of the gut whereas others, like *Helicobacter hepaticus* were increased only in the cecum. Potential beneficial organisms including *Roseburia* were decreased in the proximal regions of the TRAG colon, while *Bifidobacterium pseudolongulum* was decreased in the TRAG distal colon. Thus, the innate immune system maintains a structured, spatially organized, gut microbiome along the transverse and longitudinal axis of the gut, and disruption of this biogeography is a feature of innate immune colitis.

## Introduction

Inflammatory bowel disease (IBD) involves uncontrolled inflammation mediated by both the adaptive and innate immune systems. Innate immunity shapes the adaptive response, and vice versa, and this reciprocal interaction initiates, amplifies, and regulates inflammation. Specific contributions of innate immunity to the pathogenesis of IBD can be explored using models of colitis lacking adaptive immunity. We have previously described an innate model of IBD that occurs spontaneously in the absence of adaptive immunity, is early onset, and 100% penetrant.^1^ Specifically, mice expressing TNFAIP3 (A20) in intestinal epithelial cells (villin-TNFAIP3 mice) crossed to RAG1^−/−^ mice (villin-TNFAIP3 x RAG1^−/−^ mice; aka TRAG mice) spontaneously develop colitis that is not observed in villin-TNFAIP3 or RAG1^−/−^ littermates.^1^ We have explored the colitis in TRAG mice to better understand contributions of innate immunity to IBD.

The gut microbiome is very likely to be an important environmental trigger for IBD. Many mouse models of colitis require the presence of gut bacteria, including innate models of IBD.^2–9^ However, not all innate models of IBD require intestinal microbes for colitis.^10^ It is becoming clear that pathogenic changes in the microbiome likely extend beyond changes or differences in overall microbial populations, and that microbial functions and localization can be key determinants of pathogenic responses by the immune system.^11–17^ Differences in the localization of microbes, referred to as microbial biogeography, have been implicated in IBD but are incompletely understood, especially in the context of innate immunity.^12,18–21^ The biogeography of microbes in the colon has both cross-sectional and longitudinal features. Cross sectional biogeography includes the spatial separation of microbes from the intestinal epithelium.^12,18,20^ This is due largely to layers of mucus that exist between the epithelium and microbes in the lumen.^22^ The physical separation of epithelial and bacterial cells is also supported by anti-microbial factors and IgA found in the mucus layer and the gut lumen.^22^ The biogeography of microbes along the longitudinal axis of the colon reflects changes in oxygen tension, the proximal to distal movement of stool, nutrient availability, and other factors.^12,20^ The structured biogeography of gut microbes likely plays a role in intestinal health, and its disruption may be a feature of the pathogenesis of IBD.^23–25^

We have previously shown that villin-TNFAIP3 mice exhibit invasion of the inner mucus layer of the colon, and that villin-TNFAIP3 × IL10^−/−^ mice have early onset 100% penetrant colitis.^26^ This suggests that expression of TNFAIP3 in intestinal epithelial cells changes the cross-sectional biogeography of the microbiome, allowing colitogenic microbes to invade the inner mucus layer. The identity of the invading microbes in these mice has not been determined. TRAG mice develop colitis that is dependent on the presence of the gut microbial flora but the biogeography of microbes in this model has not been examined.^1^ Here we show that a subset of microbes, mainly Gamma proteobacteria and Actinobacteria invade the inner mucus layer of TRAG mice. In addition, the structured longitudinal biogeography in the colon of RAG1^−/−^ mice along the proximal to distal gut was disrupted and largely absent in TRAG mice. These findings indicate that the innate immune system supports a structured biogeography of the gut microbiome and that disruption of this structure is a feature of innate immune mediated colitis.

## Results

**Figure 1.**
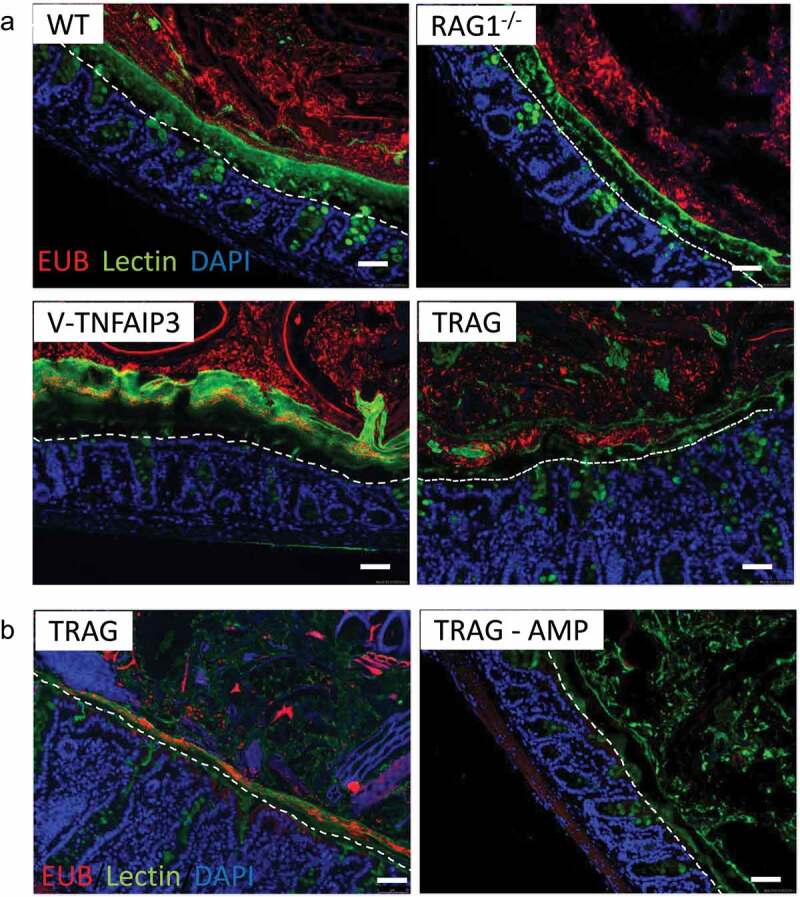
Microbes invade the mucus layer of mice expressing TNFAIP3 in intestinal epithelial cells. Colon tissues were fixed in Methyl-Carnoys, sectioned, and probed for microbes, mucus, and cells. (a) Immunolocalization of microbes (red; EUB 16S rRNA FISH), mucus (green; lectin) and colon cells (blue; DAPI) with the epithelial surface approximated by a dotted line. Microbes are present in the mucus layer of villin-TNFAIP3 (v-TNFAIP3) or villin-TNFAIP3 x RAG1^−/−^ (TRAG) mice, but less evident in WT or RAG1^−/−^ mice. (b) Mucus production in TRAG mice is not defective and normal mucus thickness is present in TRAG mice treated with antibiotic (AMP; ampicillin 0.5 g/L in drinking water, 2 weeks). Scale bar = 50uM.

TRAG mice develop colitis with 100% penetrance by 6–8 weeks of age.^1^ This colitis is prevented by treatment of TRAG mice with a cocktail of antibiotics (ampicillin, metronidazole, neomycin, and vancomycin (AMNV)), indicating the microbes drive this innate immune mediated colitis.^1^ There is a thick mucus layer in the colon that separates the large numbers of lumenal microbes from the surface of intestinal epithelial cells.^27^ In villin-TNFAIP3 transgenic mice, this mucus layer is invaded by microbes.^26^ We therefore investigated the localization of microbes, mucus, and epithelial cells in TRAG colons. The mucus layer of WT mice, visualized by lectin staining, was largely devoid of microbes which were abundant in the area overlying the mucus (Figure 1a). This spatial separation between microbes and epithelial cells by a layer of mucus was also evident in the colon of RAG1^−/−^ mice, which lack IgA and all immunoglobulins (Figure 1a). Consistent with prior results, invasion of the mucus layer by microbes was evident in the colons of villin-TNFAIP3 mice (Figure 1a). Mucosal inflammation was not observed in villin-TNFAIP3 mice, despite the invasion of the mucus layer by microbes. TRAG mice also exhibited invasion of the mucus by microbes and this was accompanied by colitis in these mice (Figure 1a). To determine whether TRAG mice have a primary defect in mucus production we reduced microbe numbers by antibiotic treatment. Treatment of TRAG mice with ampicillin restored the mucus layer, which was largely devoid of microbes (Figure 1b). Thus, innate immune mediated colitis occurs when there is the combination of microbial invasion of the colonic mucus layer concurrent with immunodeficiency (TRAG mice), but not when either microbial invasion or immunodeficiency are present independently.

**Figure 2.**
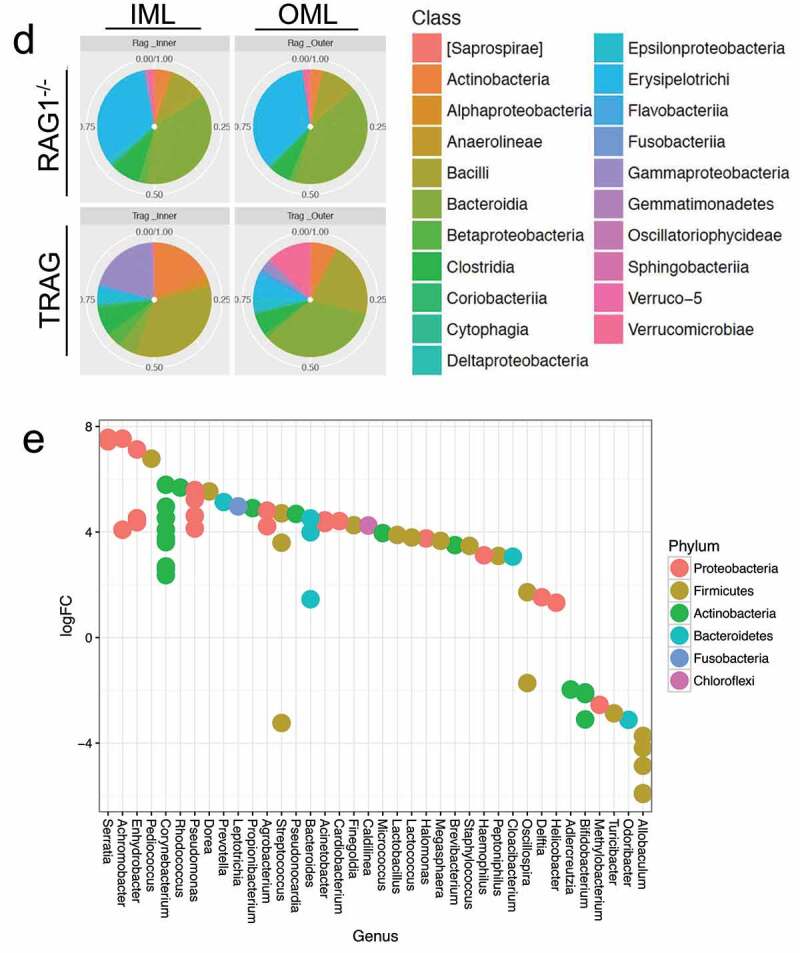
(Continued).

To identify microbes that invade the mucus layer of TRAG mice, inner and outer mucus layers from histological sections of RAG1^−/−^ and TRAG mice were obtained by laser capture microdissection and analyzed by 16S rRNA sequencing. Principal coordinate analysis using Bray-Curtis and Weighted Unifrac distances were used to compare RAG1^−/−^ inner mucus layer (IML), RAG1^−/−^ outer mucus layer (OML) to the IML and OML of TRAG mice. The IML and OML revealed differences between genotypes (TRAG v. RAG1^−/−^) with the TRAG IML having distinct differences from either mucus layer of RAG1^−/−^ mice (Figure 2a). Heat maps of OTUs suggested reduced overall diversity in the composition of TRAG mucus layers compared to RAG1^−/−^ mucus layers (Figure 2b). The composition of the TRAG IML community revealed reduced abundance of the phylum Bacteroidetes with increased abundance of the phyla Proteobacteria and Actinobacteria (Figure 2c). At the class level, TRAG IML exhibited increased Gammaproteobacteria and Actinobacteria (Figure 2d). When grouped by genotype alone, genera of Proteobacteria with increased abundance in TRAG mucus included *Serratia, Pseudomonas, Acinetobacter*, and *Cardiobacterium*, as well as some organisms capable of colonizing immunocompromised hosts including, *Achromobacter* and *Agrobacterium* (Figure 2e). Genera within Actinobacteria that were altered in TRAG mucus included *Corynebacterium* (increased) and *Bifidobacterium* (decreased) (Figure 2e). Together these findings indicate that TRAG mice exhibit altered cross-sectional biogeography in their mucus layer with increased prevalence of microbes from the Phyla Proteobacteria and Actinobacteria invading the inner mucus layer.
**Figure 2.**
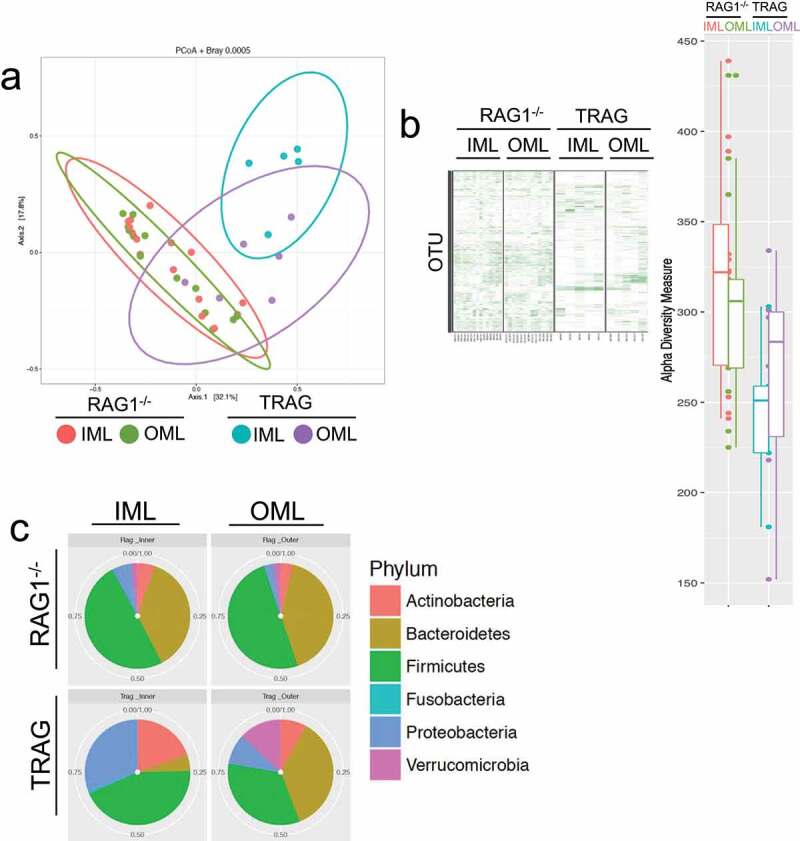
Gammaproteobacteria and Actinobacteria invade the inner mucus layer of TRAG mice. Laser capture dissection was used to collect microbial samples from Methyl-Carnoys fixed histological sections of the colon. Microbe populations in the mucus layers were characterized by 16S rRNA sequencing. (a) PCoA plots of microbe populations from RAG1^−/−^ or TRAG mucus layers (IML = inner mucus layer; OML outer mucus layer). (b) Heat maps of OTUs and Shannon diversity index from RAG1^−/−^ and TRAG IML and OML showing reduced abundance of OTUs in TRAG layers. (c) Relative abundance of Phyla in the IML and OML or RAG1^−/−^ and TRAG mice showing reduced Bacteroidetes and increased Proteobacteria and Actinobacteria especially in the IML of TRAG mice. (d) Relative abundance of microbial classes in the mucus layers of RAG1^−/−^ and TRAG mice reveals increased abundance of Gammaproteobacteria and Actinobacteria in the IML of TRAG mice. (e) Genera that are increased in the IML of TRAG mice include *Serratia, Pseudomonas, Acinetobacter* and *Cardiobacterium* (Proteobacteria) and *Corynebacterium* (Actinobacteria). TRAG: n = 6; RAG1^−/−^: n = 12.

**Figure 3.**
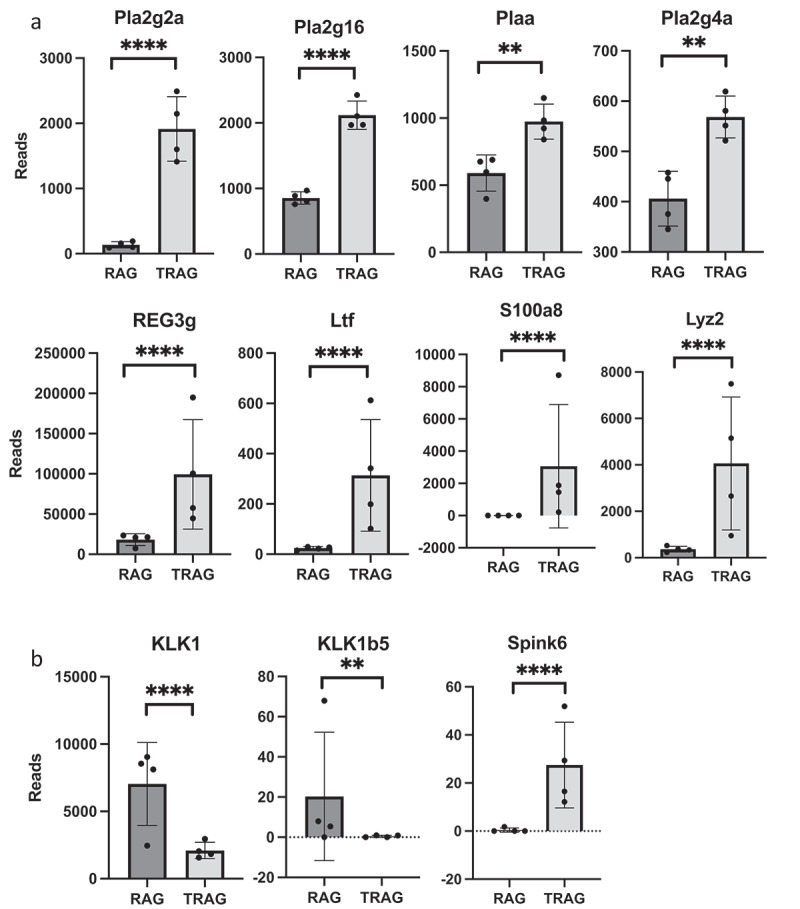
Expression of antimicrobial factors is increased in the mucosa of TRAG mice. RNA collected from mucosal scrapings of RAG1^−/−^ and TRAG mice was analyzed and quantified by RNAseq. (a) Increased expression of phospholipases (Phopholipase A2 groupIIA (PLA2g2A), Phopholipase A2 group16 (PLA2g16), PLAA (phospholipase A2 activating protein), PLA2g4a (phospholipase A2 group IVA)), Regenerating Family member 3 gamma (REG3g), lactoferrin (Ltf), calprotectin component S100A8, and lysozyme (Lyz2). (b) Decreased expression of kallikrein 1 (KLK1) and KLK1b5 and increased expression of the KLK inhibitor Spink6 (Serine peptidase inhibitor kazal type 6) in the mucosa of TRAG vs. RAG1^−/−^ mice. n = 4 **p < .01; ****p < .001.

One possible explanation for the invasion of the TRAG mucus layer by a subset of microbes might be the suppression, in TRAG colons, of antimicrobial factors that normally inhibit mucus invasion by that subset of microbes. We therefore examined the expression of known antimicrobial factors using RNAseq of mucosal scrapings from RAG^−/−^ vs. TRAG mice. Almost all antimicrobial factors were either unchanged or significantly increased in TRAG colons, including REG3γ, S100a8 (calprotectin), lactotransferrin, lysozyme 2, and secreted phospholipases (Figure 3a) which are known to have antimicrobial effects.^28^ Lastly, some kallikrein-related peptidases (KLK1, KLK1b5) were decreased in the mucosa of TRAG mice, while the KLK inhibitor SPINK6 was increased (Figure 3b). KLKs are serine proteases that have been implicated in the processing of antimicrobial peptides, and the reduced expression of these KLKs or increased expression of SPINK6 might contribute to invasion of the mucus layer by Proteobacteria and Actinobacteria.^29^

**Figure 4.**
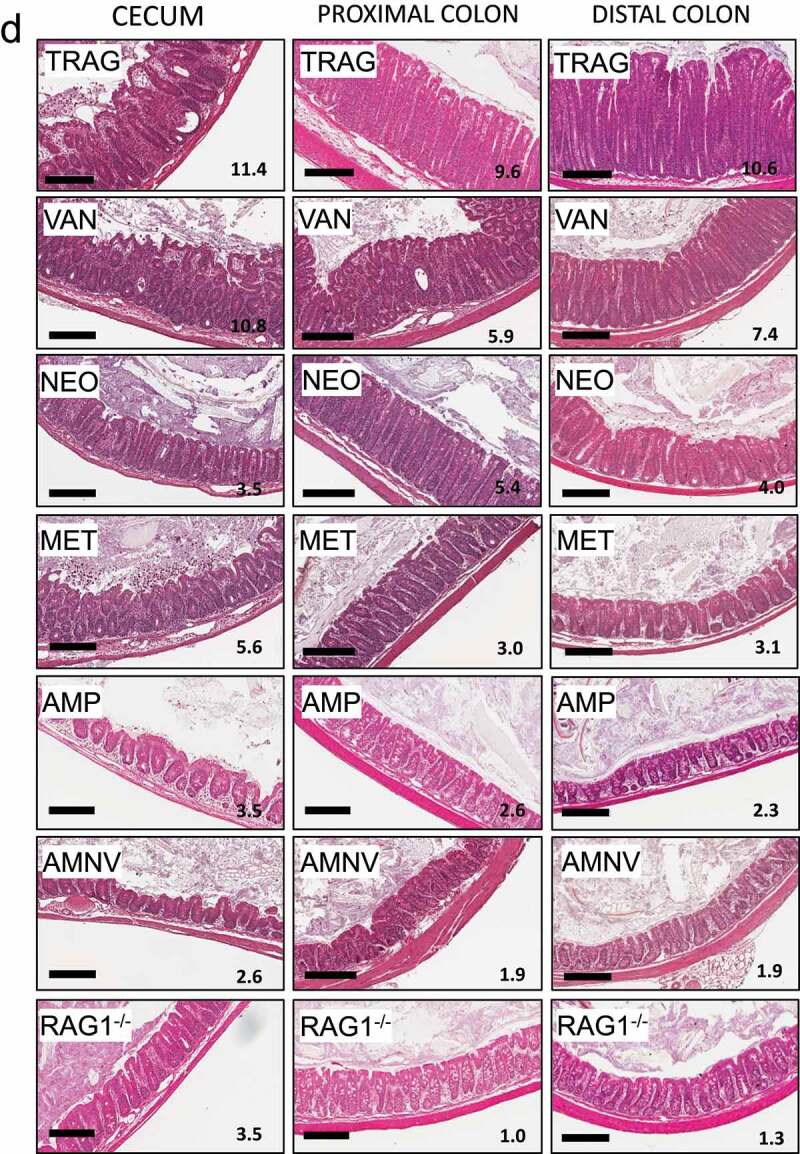
(Continued).

A cocktail of four antibiotics (AMNV) prevents colitis in TRAG mice. To better understand how different groups of bacteria might impact innate colitis, we challenged TRAG mice with each antibiotic alone. Mice were treated with antibiotics for 4 weeks, beginning at 4 weeks of age, and histological sections of cecum, proximal colon, and distal colon were scored for severity of inflammation. In the cecum, colitis was significantly attenuated by ampicillin, neomycin, or metronidazole alone, but not by vancomycin alone (Figure 4a,d). In the proximal colon, significant attenuation of colitis was achieved with either ampicillin or metronidazole, but not with vancomycin or neomycin alone (Figure 4b,d). Finally, in the distal colon, significant attenuation of colitis was achieved with ampicillin, neomycin, or metronidazole alone, but not with vancomycin (Figure 4c,d). The relative ability of individual antibiotics to suppress colitis varied along the proximal to distal axis of the gut. In the cecum, neomycin was the most effective inhibitor of inflammation, whereas neomycin did not significantly reduce inflammation in the proximal colon. Along the proximal to distal axis of the colon, the relative inhibition of inflammation by individual antibiotics was neomycin > ampicillin > metronidazole ⋙vancomycin (cecum), ampicillin > metronidazole ≫neomycin and vancomycin (proximal colon) and ampicillin > metronidazole > neomycin ⋙ vancomycin (distal colon). Thus, there are differences in effectiveness of antibiotics along the proximal to distal axis of the gut.
**Figure 4.**
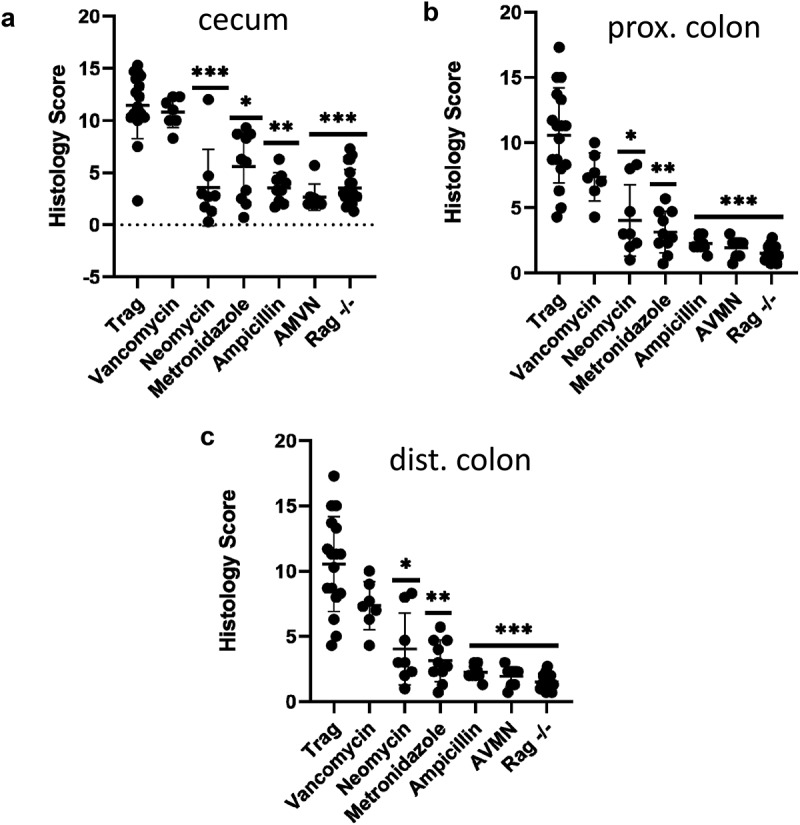
Antibiotics differentially prevent colitis in TRAG mice. Colitis scores of the (a) cecum, (b) proximal colon, or (c) distal colon of TRAG mice treated with drinking water containing vancomycin (125 mg/ml), neomycin (250 mg/ml), metronidazole (62.5 mg/ml) ampicillin (500 mg/ml) or a cocktail of all four antibiotics (AVMN). (d) representative H&E sections of TRAG cecum, proximal colon and distal colon treated with antibiotics as described. n = 8–21 *p < .05, **p < .01, ***p < .005 vs. TRAG score.

**Figure 5.**
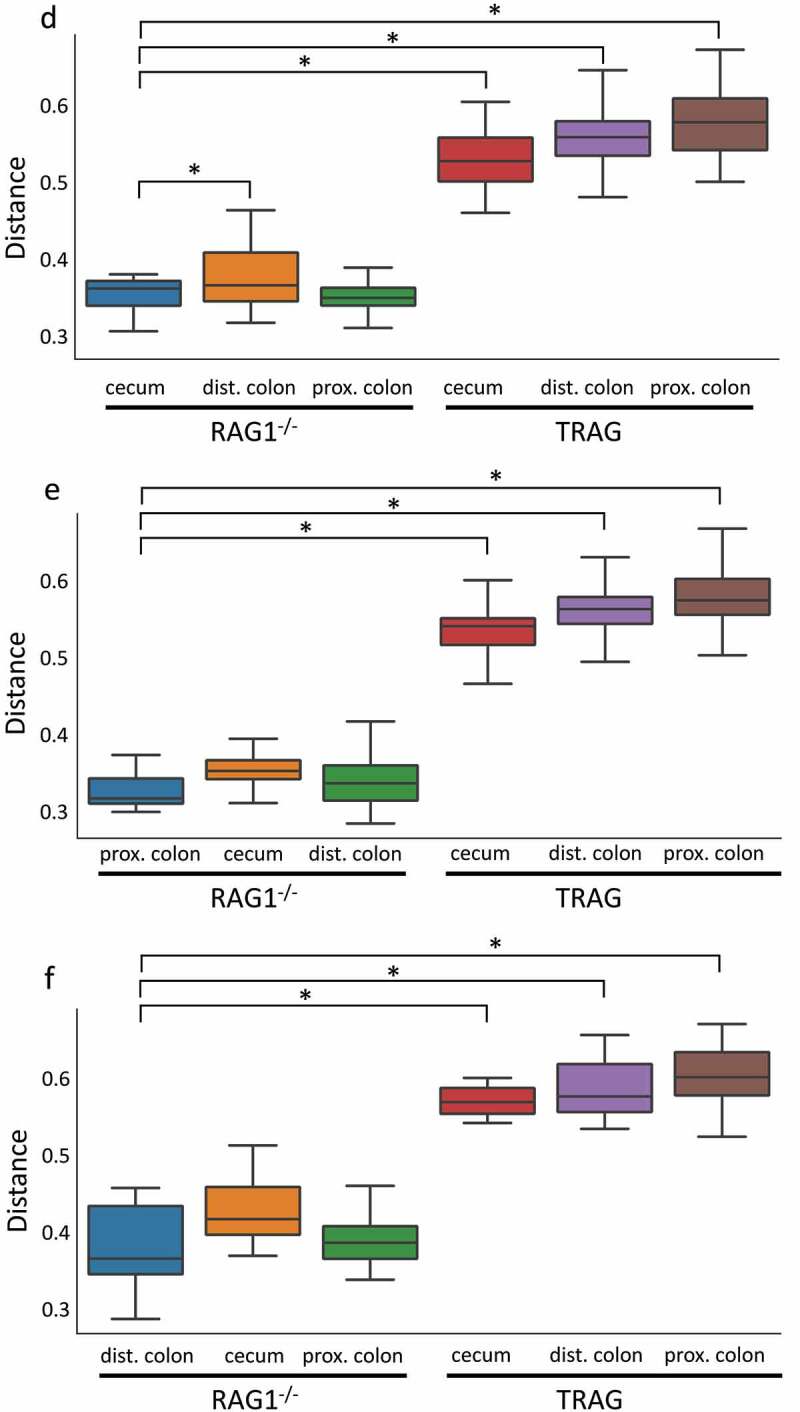
(Continued).

**Figure 6.**
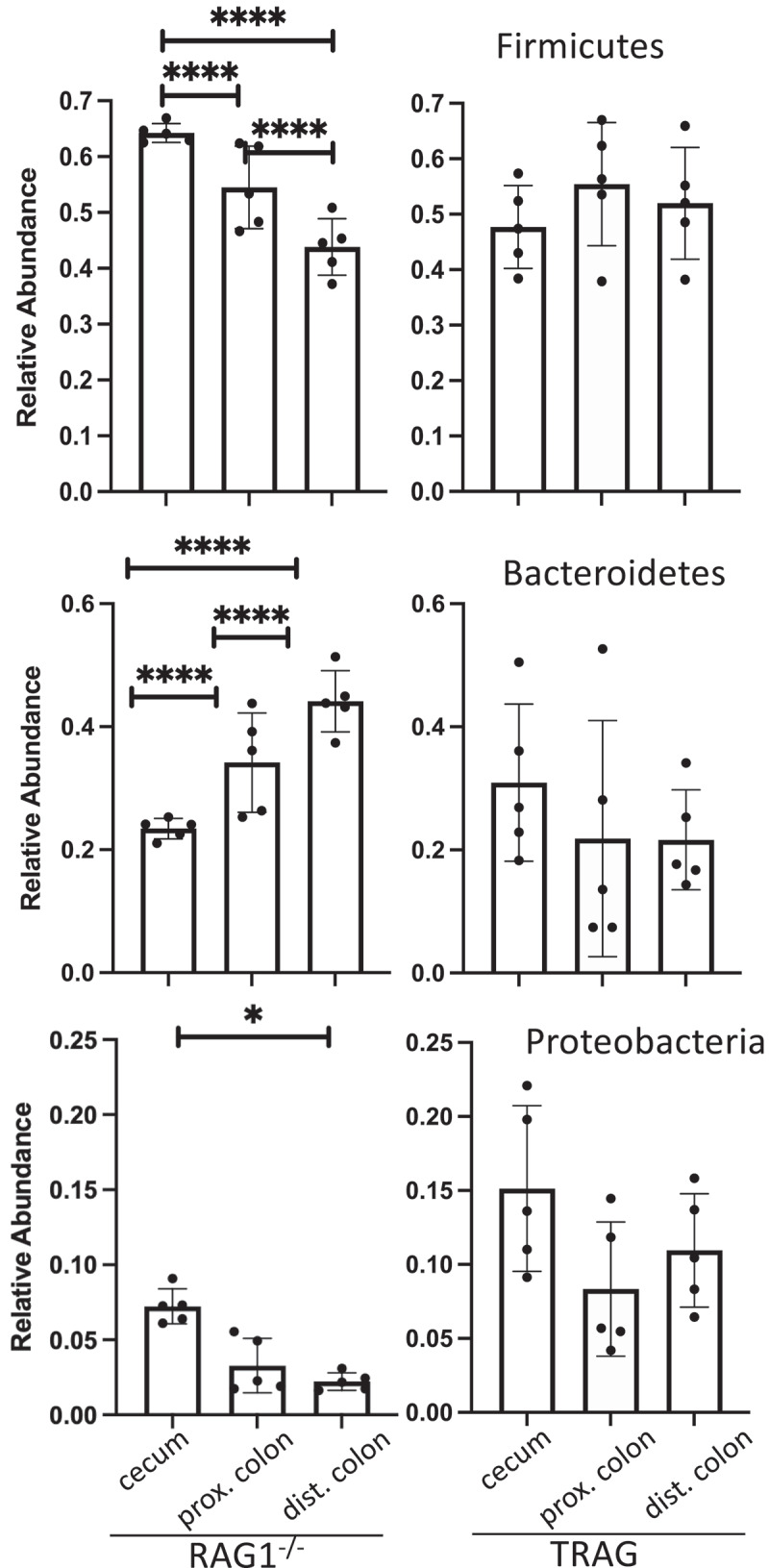
Relative abundance of the Phyla Firmicutes, Bacteroidetes and Proteobacteria across the longitudinal axis of the colon of RAG1^−/−^ or TRAG mice. n = 5 *p < .05, ****p < .001.

One potential explanation for the differences in antibiotic effectiveness along the proximal axis of the gut might be differences in proximal/distal biogeography of microbes in TRAG mice. We therefore analyzed the microbiome of RAG1^−/−^ and TRAG mice along the proximal distal axis. Gut contents were collected from the cecum, proximal colon, and distal colon of mice and analyzed by 16S rRNA sequencing. Principal component analysis using Bray-Curtis dissimilarity revealed distinct grouping of microbial communities between RAG1^−/−^ cecum, proximal colon, and distal colon (Figure 5a). This proximal-distal microbial community structure was lost in TRAG mice, where clear differences in communities between cecum, proximal colon, and distal colon were less evident. In the RAG1^−/−^ gut, the relative abundance of the phyla Firmicutes and Proteobacteria decreased along the proximal to distal axis, while the phyla Bacteroidetes exhibited increased abundance (Figure 6). Within the phylum Firmicutes, decreased abundance along the proximal distal axis was largely accounted for by decreased abundance of the class Clostridia (mainly Lachnospiraceae and Ruminococcaceae), while Bacilli and Erysipelotrichia abundance increased (Figure 7). Decreased Proteobacteria abundance was largely due to decreased abundance of the class Epsilonproteobacteria (mainly Helicobacteraceae) and a decrease in Deltaproteobacteria (mainly Desulfovibrionaceae) (Figure 7). Increased relative abundance of Phylum Actinobacteria along the RAG1^−/−^ colon was largely due to increased abundance of the class Actinobacteria (mainly Bifidobacteriaceae) (Figure 7). Finally, the increased abundance of the phylum Bacteroidetes along the proximal distal axis of the RAG1^−/−^ gut was due to increased abundance of the class Bacteroidia (mainly Bacteroidales) (Figure 7). This proximal to distal pattern of change in microbial populations was largely absent from TRAG colons. Unlike RAG1^−/−^ mice, where Bacteroidetes increased along the proximal distal axis of the colon, Bacteroidetes in TRAG colons decreased or was unchanged in abundance along the proximal distal axis of the colon (Fig 6, 7). The decrease in the abundance of Firmicutes observed along the proximal distal axis of RAG1^−/−^ mice was not evident in TRAG mice (Fig 6, 7). Thus, predictable changes in the proximal to distal biogeography of mice lacking adaptive immunity were lost or reversed in the context of innate immune colitis.
**Figure 5.**
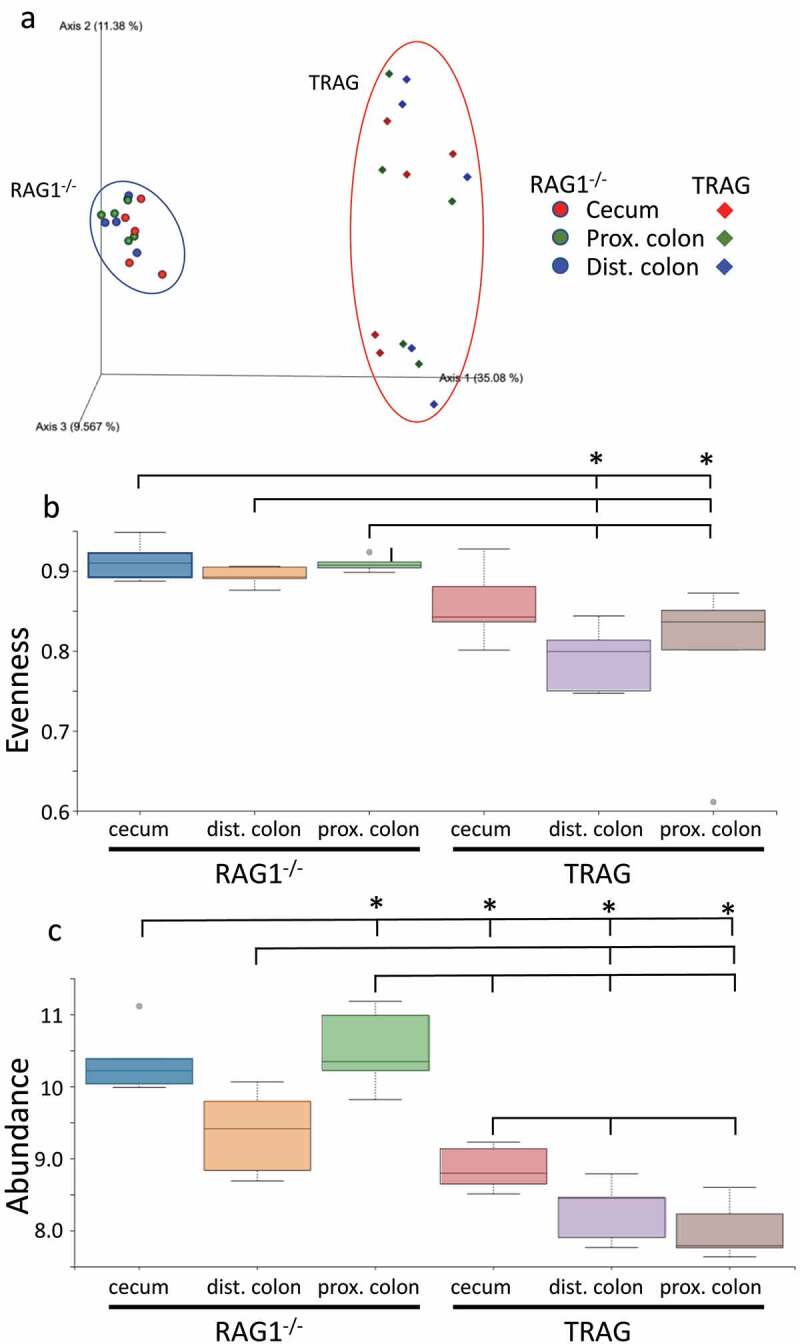
Microbiome profiles of RAG1^−/−^ vs. TRAG cecum, proximal colon and distal colon. (a) Principal component analysis (PCOoA) of beta diversity as measured by Bray-Curtis dissimilarity of RAG1^−/−^ and TRAG cecum, proximal colon and distal colon. Alpha diversity (b) Pielou’s (evenness) and (c) Faith (abundance) between groups. (d-f) Beta diversity showing distance from RAG1^−/−^ (d) cecum, (e) proximal colon and (f) distal colon. n = 5 *p < .05.

**Figure 7.**
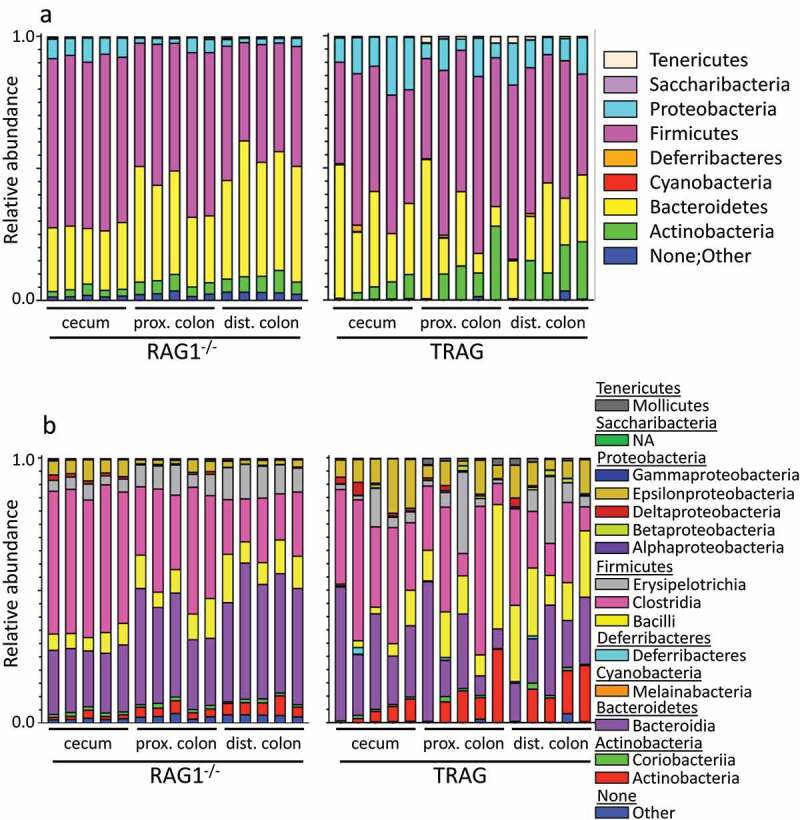
Relative abundance of microbial populations in the cecum, proximal colon and distal colon of RAG1^−/−^ and TRAG mice. (a) Phylum (b) Class and (c) Order. Each bar represents a single mouse (n = 5).

**Figure 7.**
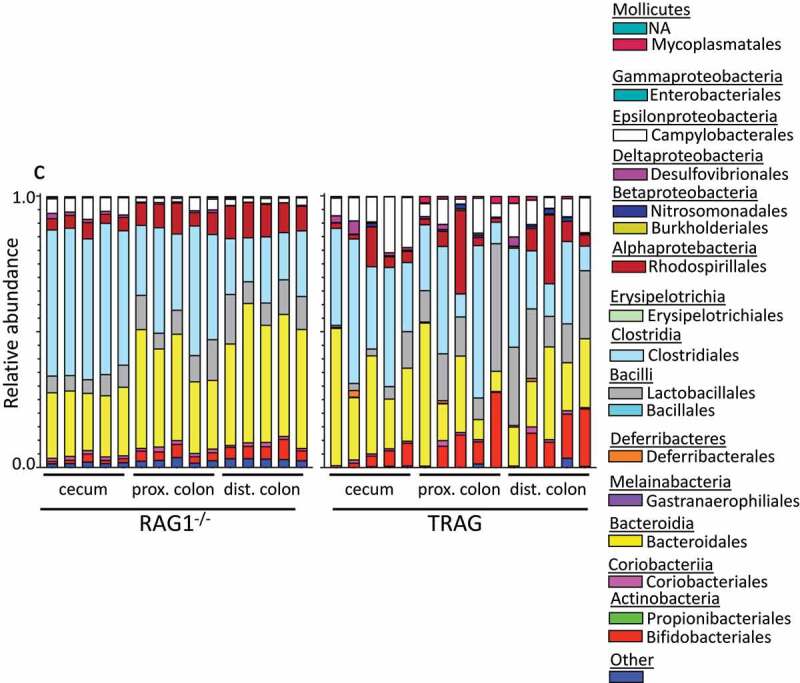
(Continued).

**Figure 8.**
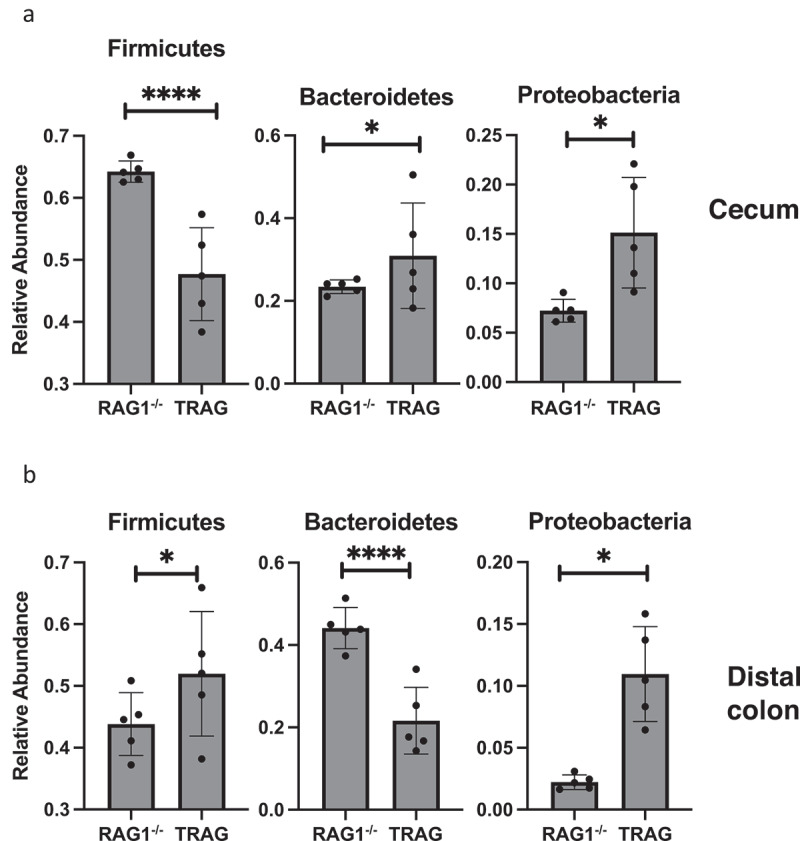
Phyla level differences in relative abundance between RAG1^−/−^ and TRAG mice. Significant differences in relative abundance were observed for the indicated Phyla in the (a) Cecum and (b) Distal colon. Only those Phyla with statistically different relative abundance are shown. No significant differences in Phyla abundance were observed in the proximal colon. n = 5 *p < .05, ****p < .001.

**Figure 9.**
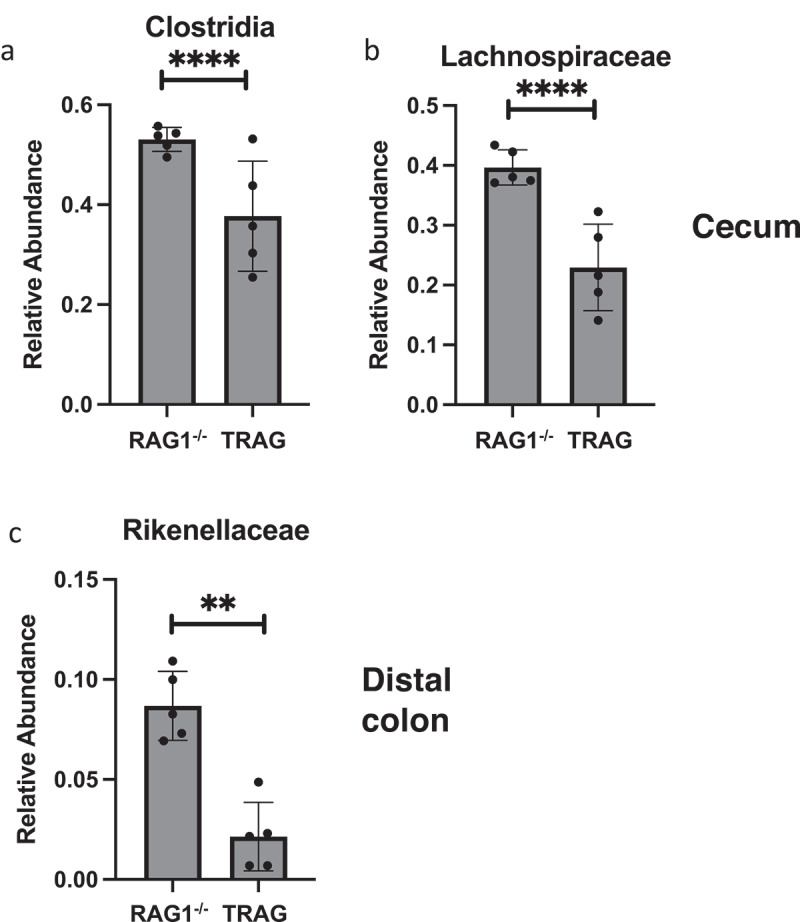
Order and Family level differences in relative abundance between RAG1^−/−^ and TRAG mice. Significant differences in relative abundance were observed for (a) the Order Clostridia only in the cecum. Differences at the Family level were observed in the (b) cecum, and (c) distal colon. Only those Orders and Families with statistically different relative abundance are shown. No significant differences in Order were observed in the proximal or distal colon and no significant differences in Family were observed in the proximal colon. n = 5 **p < .01, ****p < .001.

**Figure 10.**
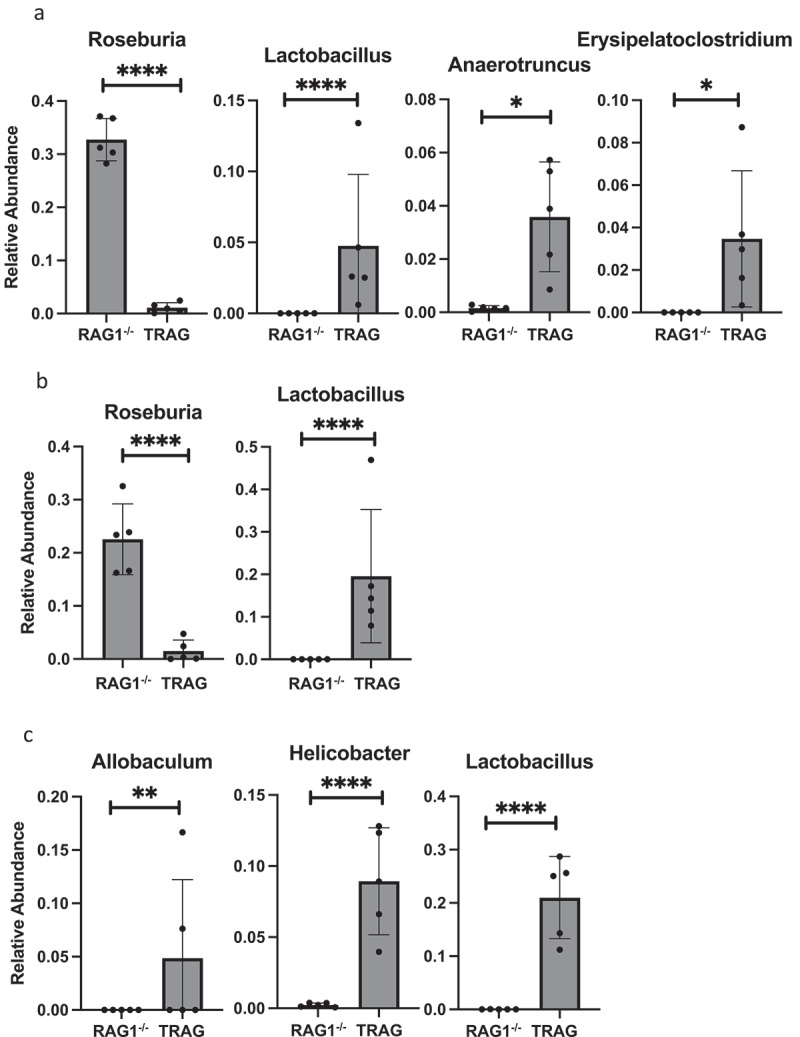
Genus level differences in relative abundance between RAG1^−/−^ and TRAG mice. Significant differences in relative abundance were observed for the indicated Genera in the (a) Cecum (b) Proximal colon and (c) Distal colon. Only those Genera with statistically different relative abundance are shown. n = 5 *p < .05, **p < .01, ****p < .001.

**Figure 11.**
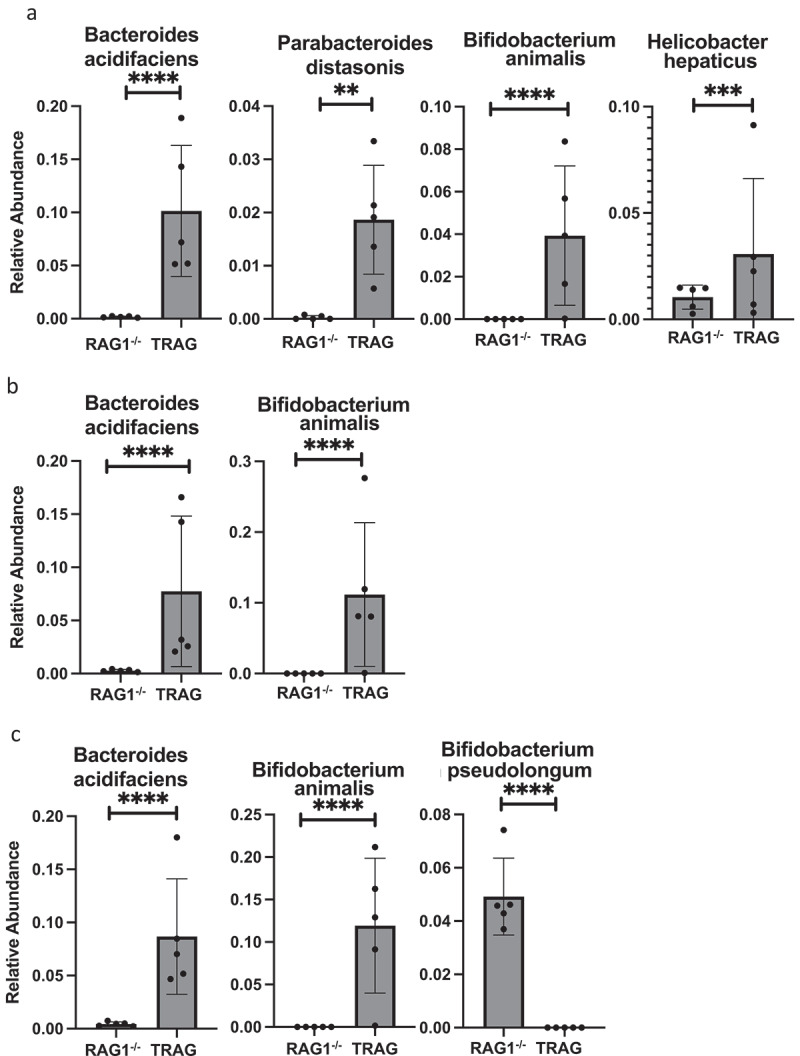
Species level differences in relative abundance between RAG1^−/−^ and TRAG mice. Significant differences in relative abundance were observed for the indicated Species in the (a) Cecum (b) Proximal colon and (c) Distal colon. Only those Species with statistically different relative abundance are shown. n = 5 **p < .01, ***p < .005, ****p < .001.

Differences in the antibiotic effectiveness along the proximal distal axis of the colon led us to compare the microbial community profiles between RAG1^−/−^ and TRAG mice in the cecum, proximal colon, and distal colon. In the cecum, reduced microbial diversity was observed in TRAG mice (Figure 5). Principal component analysis revealed dissimilarity between microbial communities of RAG1^−/−^ cecum compared to TRAG cecum (Figure 5a). The cecum of TRAG mice had lower abundance of Firmicutes and trended toward a higher abundance of Bacteroidetes, Proteobacteria, and Actinobacteria than RAG1^−/−^ mice (Fig 7, 8). Among Firmicutes, the class Clostridia, Family Lachnospiraceae and the genus *Roseburia* were reduced in the cecum of TRAG mice (Fig 7–10). The increased abundance of Bacteroidetes in the TRAG cecum included *Bacteroides acidifaciens*, and *Parabacteroides distasonis* (Figure 11). Increased Actinobacteria included *Bifidobacterium animalis* and increased Proteobacteria included *Helicobacter hepaticus* (Figure 11). Some members of the Firmicutes were also increased in abundance in TRAG cecum, including those of the genus *Lactobacillus, Anaerotruncus*, and *Erysipelatoclostridium* (Figure 10).

As in the cecum, reduced microbial diversity was observed in the TRAG proximal colon and PCoA revealed dissimilarity between the microbial communities of RAG1^−/−^ and TRAG proximal colon (Figure 5). Differences in the relative abundance of microbial species in the TRAG proximal colon were consistent with those observed in the TRAG cecum except that fewer comparisons of relative abundance reached statistical significance (Fig 6–11). The proximal colon of TRAG mice had no statistically significant changes in relative abundance of Phyla, Order, or Family (Fig 7,8). As seen in the cecum, the proximal colon of TRAG mice did have significantly reduced abundance of the genus *Roseburia* and increased abundance of the genus *Lactobacillus* (Figure 10). Additionally proximal colon of TRAG mice had increased abundance of *Bacteroides acidifaciens* and *Bifidobacterium animalis*, compared to RAG1^−/−^ mice (Figure 11).

The distal colon of TRAG mice exhibited reduced microbial diversity compared to RAG1^−/−^ mice (Figure 5). PCoA plots indicated dissimilarity between RAG1^−/−^ and TRAG distal colon (Figure 5a). Compared to RAG1^−/−^ mice, the distal colon of TRAG mice had reduced relative abundance of Bacteroidetes and increased abundance of Firmicutes (Fig 6–8). There was decreased abundance of the Families Lachnospiraceae (Firmicutes) and Rikenellaceae (Bacteroidetes) in TRAG distal colon (Figure 9). Increased abundance in the distal colon of TRAG mice included the genera *Allobaculum* and *Lactobacillus* (Firmicutes) and also *Helicobacter* (Campylobacterota) and a trend toward increased abundance of *Anaerotruncus* (Firmicutes) (p = .051; not shown) (Figure 10). As in the cecum and proximal colon, TRAG distal colon exhibited increased abundance of *Bifidobacterium animalis* and *Bacteroides acidifaciens*. TRAG distal colon also exhibited decreased abundance of *Bifidobacterium pseudolongum*, compared to RAG1^−/−^ distal colon. Together, these results indicate that the TRAG colon has less microbial diversity along the proximal to distal axis of the colon. While some microbes, such as *Bifidobacterium animalis, Bacteroides acidifaciens, Helicobacter*, and *Anaerotruncus* are increased in abundance along all regions of the TRAG colon, there are differences in the relative abundance of microbial phyla along the proximal distal axis of the colon. The relative abundance of Firmicutes was unchanged along the proximal to distal axis of the TRAG colon but decreased along the RAG1^−/−^ colon. Conversely, Bacteroides relative abundance was unchanged along the proximal to distal axis of the TRAG colon but increased along the RAG1^−/−^ colon. The relative abundance of Proteobacteria was lower in RAG1^−/−^ colons and decreased along the proximal to distal axis, whereas the TRAG colon had higher relative abundance of Proteobacteria that was unchanged along the proximal distal axis of the gut. Thus, differences in microbial populations may account for differences in antibiotic effectiveness in preventing colitis along the proximal distal axis of the colon. Additionally, the proximal to distal pattern of microbial phyla along the healthy colon of RAG1^−/−^ mice is not simply disrupted in TRAG colons but is in fact inverted.

## Discussion

Invasion of the colonic mucus layer by microbes has been implicated in inflammatory bowel disease in mice and humans.^11–13,18,20,21,23,25^ The relative paucity of microbes in the mucus layer is thought to be maintained by the physical structure of mucus and by its infusion with antimicrobial factors and sIgA.^24,30^ We observed that microbes drive innate immune mediated colitis in TRAG mice and that altered cross-sectional biogeography of microbes in the colon was evident in these mice. Specifically, microbes invade the inner mucus layer of TRAG mice that develop colitis. Microbial invasion was observed in villin-TNFAIP3 mice, which do not develop colitis. Microbial invasion was not evident on RAG1^−/−^ mice, which also do not develop colitis. This suggests that the combination of both microbial invasion and immunodeficiency is required for the innate colitis we observe in TRAG mice. In immunocompetent mice, the mucosa is enriched in IgA which coats microbes, including colitogenic microbes, to promote tolerance and gut health.^31,32^ Indeed, several members of the gut microbial community, including *Bacteroides fragilis, Blautia sp*., and Rikenellaceae may use IgA to facilitate colonization of the mucus layer.^33^ The fact that RAG1^−/−^ mice maintain a relatively microbe-free mucus layer indicates that the absence of IgA does not overtly increase invasion of the colonic mucus by gut microbes. This is consistent with prior studies of the absence of microbial invasion of the mucus layers in the context of reduced IgA.^34,35^ RAG1^−/−^ or immunodeficient mice are known to have a more robust innate immune response to colonization with microbes.^36–38^ Thus, TRAG mice might develop colitis because the RAG1^−/−^ immune system is primed to a more robust immune response to microbes and expression of TNFAIP3 in the epithelium facilitates microbial invasion. It is not clear why the expression of TNFAIP3 facilitates microbial invasion of the mucus layer. One possibility is that TNFAIP3 inhibits expression of an antimicrobial factor or factors that normally keeps the mucus layer relatively microbe free. Our RNAseq results, comparing RAG1^−/−^ and TRAG colons did not identify reduced expression of known antimicrobial factors in TRAG mice. Indeed, most known antimicrobials were either markedly induced or unchanged in TRAG vs. RAG1^−/−^ colons. Induction of antimicrobials in TRAG colons may be a response to the presence of microbes in the mucus layer, either through expression by epithelial cells or infiltration of the tissue with innate cells expressing antimicrobial factors. There may be unknown antimicrobial factors that are downregulated in the TRAG colon and these may be especially important for preventing invasion by microbes in the classes Gammaproteobacteria and Actinobacteria, as these were over-represented in the mucus layer of TRAG mice. One possible family of antimicrobial factors that might be reduced in TRAG colons are kallikrein-related peptidases (KLK). Members of this family of serine proteases are known to mediate cleavage of cathelicidin to produce the peptide LL-37 which is antimicrobial to members of the classes Gammaproteobacteria and Actinobacteria.^29,39,40^ We observed reduced expression of, KLK1 and KLK1b5 and increased expression of the KLK inhibitor Spink6 in the colon of TRAG mice, compared to RAG1^−/−^ mice.^41^ The regulation of antimicrobial peptides in the colon by KLKs or Spink6 has not been reported and further studies will be needed to determine whether suppression of antimicrobial factors mediates the microbial invasion of mucus in villin-TNFAIP3 or TRAG mice.

We observed altered cross-sectional microbial biogeography, involving increased abundance of Proteobacteria and Actinobacteria in the mucus layer, concurrent with colitis in immunocompromised mice. Encroachment of microbes into the mucus layer has been observed in human IBD patients and mouse models of IBD.^11,12,20^ Elevated Proteobacteria and Actinobacteria have been observed in IBD patients and these phyla contain numerous potential pathobionts that have the potential to cause intestinal inflammation.^11,42^ Notably, the inner mucus layer of TRAG mice exhibited elevated abundance of *Serratia*, a genus that has been implicated as a pathobiont in IBD.^43^ Although *Akkermansia muciniphila* has been identified as an important mucin-degrading microbe, we did not observe increased abundance of this organism in the mucus layers of TRAG mice.^44^ However, recent studies have expanded the potential repertoire of mucin-degrading microbes in the gut, including members of the Bacteroidetes, Actinobacteria, and Proteobacteria, some of which (*Bacteroides, Bifidobacterium, Serratia*) were found in increased abundance in TRAG mice.^45^ Changes in cross-sectional gut microbial biogeography have been implicated in colon cancer, diabetes, IBD, and other intestinal diseases.^11^ It remains to be determined whether microbial invasion of the mucus layer is a cause or a result of IBD. Our current model is that invasion of microbes into the mucus layer of TRAG mice is a cause of innate immune-mediated IBD. We observed microbial invasion of the mucus layer in healthy villin-TNFAIP3 mice which do not develop colitis but do exhibit increased FoxP3^+ve^ Tregs in their mucosa.^26^ These Foxp3^+ve^ Tregs might be preventing colitis from occurring in villin-TNFAIP3 mice, and the lack of these cells in TRAG mice might therefore contribute to the spontaneous development of innate immune mediated IBD we observe in TRAG mice.

We observed changes along the proximal to distal axis of the colon in RAG1^−/−^ mice that were absent or significantly altered in TRAG colons. RAG1^−/−^ mice exhibited increase microbial diversity, compared to TRAG mice, in all three regions of the colon (cecum, proximal colon, and distal colon). Differences in microbial populations along the proximal to distal axis of the colon have been described in rodents and humans and alterations in these patterns are found in IBD.^11,12,20,46^ The spatial structure of the microbiome in the gut likely reflects differences in pH, oxygen, antimicrobials, digesta composition, and transit times along the proximal to distal axis. Galvez et al found that WT and RAG2^−/−^ mice had similar microbial populations along the proximal to distal colon in SPF conditions, but that introduction of pathobionts (*Helicobacter, Desulfovibrio*) led to expansion of these organisms in the colon of RAG2^−/−^ mice compared to WT mice.^47^ This suggests that the presence of adaptive immune cells, and possibly IgA, is dispensable for shaping the colon microbiome in the absence of pathobionts, but that IgA may be critical for controlling pathobiont abundance when these organisms are present, as has been reported in models of IgA deficiency.^37,38,47,48^

From the cecum to the distal colon of RAG1^−/−^ mice, we observed decreased abundance of Firmicutes and Proteobacteria, and increased abundance of Bacteroidetes and Actinobacteria. These differences were largely absent in TRAG mice, and indeed TRAG mice displayed the opposite pattern in abundance of Firmicutes and Bacteroidetes with increased abundance of Firmicutes and decreased abundance of Bacteroidetes along the proximal to distal axis of the colon, compared to RAG1^−/−^ mice. Several potential pathobionts were increased in TRAG colons, including *Bifidobacterium animalis*, which is colitogenic in IL0^−/−^ mice.^49^ *H. hepaticus* has been implicated as a colitogenic organism in innate models of colitis and consistent with this, we observed increased abundance of *H. hepaticus* in regions of the TRAG colon.^50–52^ In addition, organisms that are thought to confer protection from colitis, including Lachnospiraceae and *Roseburia*, and *Bifidobacterium pseudolongum* were reduced in abundance in the colons of TRAG mice.^42,53–56^ Organisms such as *Roseburia* and other Lachnospiraceae are important producers of short chain fatty acids like butyrate which are protective and necessary for gut health and epithelial cell homeostasis.^57^ Thus, the reduced abundance of these organisms in TRAG mice may account for a failure to regulate inflammation in the colon. The combination of increased abundance of pathobionts and decreased abundance of protective organisms might combine to drive the microbe-driven colitis in this innate model. However, a role for some pathobionts and beneficial microbes in driving the colitis of TRAG mice may not be straightforward. Bifidobacteriaceae and fermented milk products from *B. animalis* ssp. Lactis are protective in the innate TRUC model of IBD.^58^ Some organisms that are potentially protective against intestinal inflammation, including *Parabacteroides distasonis* and *Bacteroidetes acidifaciens* exhibited increased abundance in regions of the TRAG colon, compared to the RAG1^−/−^ colon.^59–61^ The pathophysiology of innate colitis in TRAG mice is distinct from other innate immune models of IBD. The colitis in TRUC mice is transmissible and markedly reduced in C57Bl/6 mice, whereas TRAG colitis is not transmissible and is highly penetrant and early onset in C57Bl/6 mice.^2,62^ Innate colitis in RAG^−/−^ mice induced by anti-CD40 treatment or *H. hepaticus* infection is acute, systemic, and driven by ILC3 cells which is not the case for TRAG mice.^52^ Thus, differences in the gut microbiome or response to the microbiome between these innate models of IBD may reflect differences in the nature of the innate inflammation or the response of the innate system to gut microbes.

It is clear that microbes drive the innate immune colitis observed in TRAG mice. The altered biogeography of microbes and differential responses to antibiotics suggest that a subset of bacteria, especially ampicillin-sensitive microbes, are pathogenic in this model. We do not have detailed information from the current studies regarding how antibiotics alter the microbiome along the cross-sectional or longitudinal axis of the gut. Future studies, especially those to identify ampicillin sensitive colitogenic microbes in this model, will help to define innate immune responses capable of contributing to IBD.

## Methods and materials

All methods were carried out in accordance with relevant guidelines and regulations. The ARRIVE 2.0 guidelines were followed.

### Animal studies

Mice were bred and housed in the Freimann Life Sciences Center at the University of Notre Dame. Previously, these mice were generated, bred, and housed at the University of Chicago animal care facility.^63^ All protocols were performed at the University of Notre Dame Freimann Life Science Center approved by Institutional Animal Care and Use Committees. All mice were bred and maintained on the C57BL/6 background. The villin-TNFAIP3 strain was generated previously using BAC-recombineering of the villin locus and characterized as described.^63^ RAG1^−/−^ mice (C57Bl/6) were purchased from Jackson Laboratories and interbred to villin-TNFAIP3 mice to generate villin-TNFAIP3 × RAG1^−/−^ (TRAG) mice or RAG1^−/−^ littermate controls.

### Antibiotic treatments

At four weeks of age mice were treated with an antibiotic cocktail that contained vancomycin 125 mg/L, neomycin 250 mg/L, metronidazole 62.5 mg/L, and ampicillin 500 mg/L. These antibiotics were dissolved in Grape Kool-Aid. Mice were treated for four weeks then euthanized at eight weeks of age. Control mice were treated with Grape Kool-Aid without antibiotics for the same duration of time. Subsequent experiments investigating the role of each individual antibiotic were started at four weeks of age and administered for four weeks, with mice euthanized at eight weeks of age. Individual antibiotics were given as vancomycin 125 mg/L, neomycin 250 mg/L, metronidazole 62.5 mg/L, or ampicillin 500 mg/L, respectively, in Grape Kool-Aid.

### Localization of bacteria (FISH) and mucus (lectin)

RAG1^−/−^, TRAG, WT, and A20 Transgenic mice were sacrificed at eight weeks of age and colons were fixed (18 hr, RT) in Methyl Carnoy’s (60% methanol, 30% chloroform, 10% acetic acid), embedded in paraffin and cut in 5uM sections, as decribed.^64^ Sections were dewaxed, rehydrated, and incubated at 50°C overnight in hybridization buffer (20 mM Tris–HCl, pH 7.4, 0.9 M NaCl, 0.1% SDS, 35% (v/v) formamide) containing a fluorescently tagged Alexa Fluor 660-conjugated bacterial 16S rRNA targeted probe (EUB338; 5’-GCTGCCTCCCGTAGGAGT-3’; IDT Inc, Coralville, CA))[REF]. Slides were washed in sodium citrate buffer (3 M Nacl, 0.3 M sodium citrate, pH 7.0) and then washed in PBS to prepare for localization of mucus. Slides were incubated (1 hr, RT) in blocking buffer (5% serum, 1%BSA in TBS-T (19 mM Tris base, 2.7 mM KCl, 137 mM NaCl, 0.05% (v/v) Tween-20)), washed, and incubated (2 hr, RT) with Alexa-Fluor 488 conjugated lectin (GeneTex GTX01512) in blocking buffer. Slides were washed in PBS, counterstained with DAPI, and mounted using prolong gold antifade reagent (Invitrogen). Slides were imaged using a Leica DM5500 B fluorescent microscope.

### RNA extraction and RNAseq analysis

Tissues were harvested and immediately frozen in liquid nitrogen and stored at −80°C. RNA was extracted using TRIzol reagent (Thermo Fisher) as per the manufacturer protocol and stored at −80°C. Preparation of RNA library and transcriptome sequencing was conducted by Novogene Co., LTD (Sacramento, CA).

Analysis of RNAseq was performed according to the methods provided by Novogene as follows: Raw data (raw reads) of fastq format were firstly processed through in-house perl scripts. In this step, clean data (clean reads) were obtained by removing reads containing adapter, reads containing ploy-N and low-quality reads from raw data. At the same time, Q20, Q30, and GC content the clean data were calculated. All the downstream analyses were based on the clean data with high quality.

Reference genome and gene model annotation files were downloaded from genome website directly. Index of the reference genome was built using Hisat2 v2.0.5 and paired-end clean reads were aligned to the reference genome using Hisat2 v2.0.5. We selected Hisat2 as the mapping tool for that Hisat2 can generate a database of splice junctions based on the gene model annotation file and thus a better mapping result than other non-splice mapping tools.

featureCounts v1.5.0-p3 was used to count the reads numbers mapped to each gene. And then FPKM of each gene was calculated based on the length of the gene and reads count mapped to this gene. FPKM, expected number of Fragments Per Kilobase of transcript sequence per Millions base pairs sequenced, considers the effect of sequencing depth and gene length for the reads count at the same time, and is currently the most commonly used method for estimating gene expression levels.

Differential expression analysis of two conditions/groups (two biological replicates per condition) was performed using the DESeq2 R package (1.20.0). DESeq2 provides statistical routines for determining differential expression in digital gene expression data using a model based on the negative binomial distribution. The resulting P-values were adjusted using the Benjamini and Hochberg’s approach for controlling the false discovery rate. Genes with an adjusted P-value ≤0.05 found by DESeq2 were assigned as differentially expressed.

### Zymobiomics 16S rRNA illumina sequencing analysis

Fecal samples were isolated from the cecum, proximal colon, and distal colon and immediately frozen in liquid nitrogen. The samples were processed and analyzed with the ZymoBIOMICS® Targeted Sequencing Service (Zymo Research, Irvine, CA).

### Histology scoring

Mouse intestinal tissues were fixed in Methyl Carnoy’s and stained via H & E. Sections were then scored using methods previously described.^65^ Briefly, the following eight categories were assessed on a scale of 0–3 to determine the severity of colitis: infiltrate, goblet cell loss, crypt density, crypt hyperplasia, muscle thickening, submucosal infiltrate, and presence of ulceration or abscesses to produce a score ranging from 0 to 24. Each category was assessed with the following criteria: Infiltrate: 0-none, 1-increased presence of inflammatory cells, 2-infiltrates also in submucosa, 3- transmural. Goblet cell loss: 0-none, 1- <10%, 2–10-50%, 3- >50%. Crypt density: 0- normal, 1-decreased by <10%, 2- decreased by 10–50%, 3-decreased by >50%. Crypt hyperplasia: 0-none, 1- slight increase in crypt length, 2–2 to 3-fold increase in crypt length, 3- threefold increase in crypt length. Muscle thickening: 0-none, 1-slight, 2-strong, 3- excessive. Submucosal inflammation: 0-none, 1-individual cells, 2-infiltrate, 3- large infiltrates. Crypt Abscess and ulceration was either absent (0) or present (3).

### Laser capture microscopy for bacterial identification

TRAG and RAG-1^−/−^ colon tissues from 7- to 8-week-old mice were fixed in Methyl Carnoy’s, embedded in paraffin, sectioned, deparaffinized, and hydrated. Slides were then stained with Alcian Blue (pH 2.5) for 15 min and counterstained with (0.1%) Nuclear Fast red to orient tissues for mucus layer microdissection.

Laser Capture Microscopy (Acturus XT, LCM) was used to dissect and collect the inner and outer mucus layers from the Alcian Blue stained sections onto LCM caps. DNA was extracted from LCM caps with Arcturus PicoPure DNA Isolation Kit (Thermo-Fisher) as per manufacturer’s instructions. Laser-captured sections were incubated (3 hr, 65°C) with PicoPure solution followed by inactivation of proteinases (10 min at 95°C). DNA extracted from LCM sections was frozen and sent to Argonne National Labs for bacterial 16S rRNA sequencing.

### Laser capture microscopy bacterial 16S rRNA analysis

QIIME (Version 1.9.1) software was used to analyze the raw fastq 16S rRNA sequencing data. For quality filtering, we used a Phred score of 30, removed chimeras, and removed samples with sequencing below 5000. These sequences were clustered into Operational taxonomic units (OTUs) using uclust open reference as the clustering algorithm with Greengenes reference database and a sequencing identity match of 97%. The most detailed taxonomic level was chosen for each OTU. Alpha and Beta diversity were assessed by phylogenetic diversity using Phyloseq and Vegan in R studio. Samples were grouped by genotype (TRAG and RAG1^−/−^) and inner or outer mucus layer. Shannon diversity index was used to analyze Alpha diversity. Principal coordinates analysis (PCoA) plots using Bray-Curtis and Weighted UniFrac distances were used to assess the variation between samples.

### Fecal sample microbiome 16S rRNA analysis

QIIME2 2022.2.^66^ Raw sequence data were demultiplexed and quality filtered using the q2‐demux plugin followed by denoising with DADA2 (via q2‐dada2).^67^ All amplicon sequence variants (ASVs) were aligned with mafft (via q2‐alignment) and used to construct a phylogeny with fasttree2 (via q2‐phylogeny).^68–70^ Alpha‐diversity metrics (observed features and Faith’s Phylogenetic Diversity^71^), beta diversity metrics (weighted UniFrac, unweighted UniFrac,^72,73^ Jaccard distance, and Bray‐Curtis dissimilarity), and Principle Coordinate Analysis (PCoA) were estimated using q2‐diversity after samples were rarefied (subsampled without replacement) to 900 sequences per sample. Taxonomy was assigned to ASVs using the q2‐feature‐classifier^74,75^ classify‐sklearn naïve Bayes taxonomy classifier against the Greengenes 13_8 99% OTUs reference sequences.^76^

### Statistical analysis

Each “n” represents an individual mouse for the indicated experiments.

**Histology Scoring Statistical Analysis**: Graphpad prism version 9.3.1 was used to graph and calculate statistics of histology scoring data. A one-way ANOVA was performed with a non-parametric test (Kruskal-Wallis) and a Dunn’s multiple comparisons test.

**Laser Capture Microscopy 16**S rRNA **Statistical Analysis**: ANOVAS were employed for statistical analysis for both Alpha and Beta analysis.

**Fecal Sample Microbiome 16**S rRNA **Statistical Analysis**: Graphpad Prism (version 9.3.1) was used to analyze statistical significance for microbial speciation, while alpha and beta diversity statistics were calculated using QIIME2 2022.2. Microbial speciation was compared using a two-way ANOVA with multiple comparisons (p < .05). Alpha diversity was analyzed using a Kruskal–Wallis pairwise analysis, and beta diversity was analyzed using a PERMANOVA test p < .05).

## Data Availability

The authors confirm that data supporting the RNAseq results are available in the supplementary materials associated with the manuscript. Raw data supporting the microbiome studies are available upon request from the corresponding author, DLB.

## References

[1] Overstreet AM, LaTorre DL, Abernathy-Close L, Murphy SF, Rhee L, Boger AM, Adlaka KR, Iverson AM, Bakke DS, Weber CR, et al. The JAK inhibitor ruxolitinib reduces inflammation in an ILC3-independent model of innate immune colitis. Mucosal Immunol. 2018;11(5):1454–24. doi:10.1038/s41385-018-0051-2. 29988117 PMC6162142

[2] Garrett WS, Lord GM, Punit S, Lugo-Villarino G, Mazmanian SK, Ito S, Glickman JN, Glimcher LH. Communicable ulcerative colitis induced by T-bet deficiency in the innate immune system. Cell. 2007;131(1):33–45. doi:10.1016/j.cell.2007.08.017. 17923086 PMC2169385

[3] Garrett WS, Gallini CA, Yatsunenko T, Michaud M, DuBois A, Delaney ML, Punit S, Karlsson M, Bry L, Glickman JN, et al. Enterobacteriaceae act in concert with the gut microbiota to induce spontaneous and maternally transmitted colitis. Cell Host Microbe. 2010;8(3):292–300. doi:10.1016/j.chom.2010.08.004. 20833380 PMC2952357

[4] Onderdonk AB, Franklin ML, Cisneros RL. Production of experimental ulcerative colitis in gnotobiotic Guinea pigs with simplified microflora. Infect Immun. 1981;32(1):225–231. doi:10.1128/iai.32.1.225-231.1981. 7216487 PMC350611

[5] Prescott JF, Barker IK, Manninen KI, Miniats OP. Campylobacter jejuni colitis in gnotobiotic dogs. Canadian Journal of Comparative Medicine: Revue Canadienne de Medecine Comparee. 1981;45:377–383. 7337869 PMC1320167

[6] Rogala AR, Oka A, Sartor RB. Strategies to dissect host-microbial immune interactions that determine mucosal homeostasis vs. Intestinal Inflammation in Gnotobiotic Mice. Front Immunol. 2020;11:214. 32133003 10.3389/fimmu.2020.00214 PMC7040030

[7] Sadlack B, Merz H, Schorle H, Schimpl A, Feller AC, Horak I. Ulcerative colitis-like disease in mice with a disrupted interleukin-2 gene. Cell. 1993;75:253–261. 8402910 10.1016/0092-8674(93)80067-o

[8] Sartor RB, Wu GD. Roles for intestinal bacteria, viruses, and fungi in pathogenesis of inflammatory bowel diseases and therapeutic approaches. Gastroenterology. 2017;152:327–39.e4. 27769810 10.1053/j.gastro.2016.10.012 PMC5511756

[9] Sellon RK, Tonkonogy S, Schultz M, Dieleman LA, Grenther W, Balish E, Rennick DM, Sartor RB. Resident enteric bacteria are necessary for development of spontaneous colitis and immune system activation in interleukin-10-deficient mice. Infect Immun. 1998;66:5224–5231. 9784526 10.1128/iai.66.11.5224-5231.1998 PMC108652

[10] Uhlig HH, McKenzie BS, Hue S, Thompson C, Joyce-Shaikh B, Stepankova R, Robinson N, Buonocore S, Tlaskalova-Hogenova H, Cua DJ, et al. Differential activity of IL-12 and IL-23 in mucosal and systemic innate immune pathology. Immunity. 2006;25(2):309–318. doi:10.1016/j.immuni.2006.05.017. 16919486

[11] Daniel N, Lécuyer E, Chassaing B. Host/microbiota interactions in health and diseases-Time for mucosal microbiology! Mucosal Immunol. 2021;14(5):1006–1016. doi:10.1038/s41385-021-00383-w. 33772148 PMC8379076

[12] Donaldson GP, Lee SM, Mazmanian SK. Gut biogeography of the bacterial microbiota. Nat Rev Microbiol. 2016;14(1):20–32. doi:10.1038/nrmicro3552. 26499895 PMC4837114

[13] Donaldson GP, Chou W-C, Manson AL, Rogov P, Abeel T, Bochicchio J, Ciulla D, Melnikov A, Ernst PB, Chu H, et al. Spatially distinct physiology of bacteroides fragilis within the proximal colon of gnotobiotic mice. Nat Microbiol. 2020;5(5):746–756. doi:10.1038/s41564-020-0683-3. 32152589 PMC7426998

[14] Nava GM, Stappenbeck TS. Diversity of the autochthonous colonic microbiota. Gut Microbes. 2011;2(2):99–104. doi:10.4161/gmic.2.2.15416. 21694499 PMC3225773

[15] Nava GM, Friedrichsen HJ, Stappenbeck TS. Spatial organization of intestinal microbiota in the mouse ascending colon. ISME J. 2011;5(4):627–638. doi:10.1038/ismej.2010.161. 20981114 PMC3105732

[16] Pédron T, Mulet C, Dauga C, Frangeul L, Chervaux C, Grompone G, Sansonetti PJ. A crypt-specific core microbiota resides in the mouse colon. mBio . 2012;3:3. 10.1128/mBio.00116-12 PMC3372965 22617141

[17] Saffarian A, Mulet C, Regnault B, Amiot A, Tran-Van-Nhieu J, Ravel J, Sobhani I, Sansonetti PJ, Pédron T. Crypt. and Mucosa-Associated Core Microbiotas in Humans and Their Alteration in Colon Cancer Patients. mBio. 2019;10(4). 10.1128/mBio.01315-19 PMC6635529 31311881

[18] Lavelle A, Lennon G, Winter DC, O’Connell PR. Colonic biogeography in health and ulcerative colitis. Gut Microbes. 2016;7(5):435–442. doi:10.1080/19490976.2016.1216748. 27662587 PMC5154370

[19] Sheth RU, Li M, Jiang W, Sims PA, Leong KW, Wang HH. Spatial metagenomic characterization of microbial biogeography in the gut. Nat Biotechnol. 2019;37(8):877–883. doi:10.1038/s41587-019-0183-2. 31332325 PMC6679743

[20] Tropini C, Earle KA, Huang KC, Sonnenburg JL. The gut microbiome: connecting spatial organization to function. Cell Host Microbe. 2017;21(4):433–442. doi:10.1016/j.chom.2017.03.010. 28407481 PMC5576359

[21] Lee SM, Donaldson GP, Mikulski Z, Boyajian S, Ley K, Mazmanian SK. Bacterial colonization factors control specificity and stability of the gut microbiota. Nature. 2013;501(7467):426–429. doi:10.1038/nature12447. 23955152 PMC3893107

[22] Johansson ME, Hansson GC. Immunological aspects of intestinal mucus and mucins. Nat Rev Immunol. 2016;16(10):639–649. doi:10.1038/nri.2016.88. 27498766 PMC6435297

[23] Johansson ME, Gustafsson JK, Sjöberg KE, Petersson J, Holm L, Sjövall H, Hansson GC, Ernberg IT. Bacteria penetrate the inner mucus layer before inflammation in the dextran sulfate colitis model. PLoS One. 2010;5(8):e12238. doi:10.1371/journal.pone.0012238. 20805871 PMC2923597

[24] Johansson ME, Sjövall H, Hansson GC. The gastrointestinal mucus system in health and disease. Nat Rev Gastroenterol Hepatol. 2013;10(6):352–361. doi:10.1038/nrgastro.2013.35. 23478383 PMC3758667

[25] Johansson ME, Gustafsson JK, Holmén-Larsson J, Jabbar KS, Xia L, Xu H, Ghishan FK, Carvalho FA, Gewirtz AT, Sjövall H, et al. Bacteria penetrate the normally impenetrable inner colon mucus layer in both murine colitis models and patients with ulcerative colitis. Gut. 2014;63(2):281–291. doi:10.1136/gutjnl-2012-303207. 23426893 PMC3740207

[26] Murphy SF, Rhee L, Grimm WA, Weber CR, Messer JS, Lodolce JP, Chang JE, Bartulis SJ, Nero T, Kukla RA, et al. Intestinal epithelial expression of TNFAIP3 results in microbial invasion of the inner mucus layer and induces colitis in IL-10-deficient mice. Am J Physiol Gastrointest Liver Physiol. 2014;307(9):G871–82. doi:10.1152/ajpgi.00020.2014. 25234043 PMC4216993

[27] Johansson ME, Phillipson M, Petersson J, Velcich A, Holm L, Hansson GC. The inner of the two Muc2 mucin-dependent mucus layers in colon is devoid of bacteria. Proc Natl Acad Sci U S A. 2008;105(39):15064–15069. doi:10.1073/pnas.0803124105. 18806221 PMC2567493

[28] Buckland AG, Wilton DC. The antibacterial properties of secreted phospholipases A2. Biochim Biophys Acta. 2000;1488(1–2):71–82. doi:10.1016/S1388-1981(00)00111-6. 11080678

[29] Muytjens CM, Vasiliou SK, Oikonomopoulou K, Prassas I, Diamandis EP. Putative functions of tissue kallikrein-related peptidases in vaginal fluid. Nat Rev Urol. 2016;13(10):596–607. doi:10.1038/nrurol.2016.161. 27603220

[30] Ermund A, Schütte A, Johansson ME, Gustafsson JK, Hansson GC. Studies of mucus in mouse stomach, small intestine, and colon. I. Gastrointestinal mucus layers have different properties depending on location as well as over the Peyer’s patches. Am J Physiol Gastrointest Liver Physiol. 2013;305(5):G341–7. doi:10.1152/ajpgi.00046.2013. 23832518 PMC3761247

[31] Shapiro JM, de Zoete MR, Palm NW, Laenen Y, Bright R, Mallette M, Bu K, Bielecka AA, Xu F, Hurtado-Lorenzo A, et al. Immunoglobulin A targets a unique subset of the microbiota in inflammatory bowel disease. Cell Host Microbe. 2021;29(1):83–93.e3. doi:10.1016/j.chom.2020.12.003. 33385335 PMC10477929

[32] Palm NW, de Zoete MR, Cullen TW, Barry NA, Stefanowski J, Hao L, Degnan PH, Hu J, Peter I, Zhang W, et al. Immunoglobulin A coating identifies colitogenic bacteria in inflammatory bowel disease. Cell. 2014;158(5):1000–1010. doi:10.1016/j.cell.2014.08.006. 25171403 PMC4174347

[33] Donaldson GP, Ladinsky MS, Yu KB, Sanders JG, Yoo BB, Chou W-C, Conner ME, Earl AM, Knight R, Bjorkman PJ, et al. Gut microbiota utilize immunoglobulin A for mucosal colonization. Science. 2018;360(6390):795–800. doi:10.1126/science.aaq0926. 29724905 PMC5973787

[34] Rogier EW, Frantz AL, Bruno ME, Kaetzel CS. Secretory IgA is concentrated in the outer layer of colonic mucus along with gut bacteria. Pathogens. 2014;3(2):390–403. doi:10.3390/pathogens3020390. 25437806 PMC4243452

[35] Frantz AL, Rogier EW, Weber CR, Shen L, Cohen DA, Fenton LA, Bruno ME, Kaetzel CS. Targeted deletion of MyD88 in intestinal epithelial cells results in compromised antibacterial immunity associated with downregulation of polymeric immunoglobulin receptor, mucin-2, and antibacterial peptides. Mucosal Immunol. 2012;5(5):501–512. doi:10.1038/mi.2012.23. 22491177 PMC3422608

[36] Keilbaugh SA. Activation of RegIII / and interferon expression in the intestinal tract of SCID mice: an innate response to bacterial colonisation of the gut. Gut. 2005;54(5):623–629. doi:10.1136/gut.2004.056028. 15831905 PMC1774500

[37] Peterson DA, McNulty NP, Guruge JL, Gordon JI. IgA response to symbiotic bacteria as a mediator of gut homeostasis. Cell Host Microbe. 2007;2(5):328–339. doi:10.1016/j.chom.2007.09.013. 18005754

[38] Fagarasan S, Muramatsu M, Suzuki K, Nagaoka H, Hiai H, Honjo T. Critical roles of activation-induced cytidine deaminase in the homeostasis of gut flora. Science. 2002;298(5597):1424–1427. doi:10.1126/science.1077336. 12434060

[39] Yamasaki K, Schauber J, Coda A, Lin H, Dorschner RA, Schechter NM, Bonnart C, Descargues P, Hovnanian A, Gallo RL. Kallikrein-mediated proteolysis regulates the antimicrobial effects of cathelicidins in skin. FASEB J. 2006;20(12):2068–2080. doi:10.1096/fj.06-6075com. 17012259

[40] Borgoño CA, Gavigan J-A, Alves J, Bowles B, Harris JL, Sotiropoulou G, Diamandis EP. Defining the extended substrate specificity of kallikrein 1-related peptidases. Biol Chem. 2007;388(11):1215–1225. doi:10.1515/BC.2007.124. 17976015

[41] Kantyka T, Fischer J, Wu Z, Declercq W, Reiss K, Schröder J-M, Meyer-Hoffert U. Inhibition of kallikrein-related peptidases by the serine protease inhibitor of kazal-type 6. Peptides. 2011;32(6):1187–1192. doi:10.1016/j.peptides.2011.03.009. 21439340

[42] Lepage P, Häsler R, Spehlmann ME, Rehman A, Zvirbliene A, Begun A, Ott S, Kupcinskas L, Doré J, Raedler A, et al. Twin study indicates loss of interaction between microbiota and mucosa of patients with ulcerative colitis. Gastroenterology. 2011;141(1):227–236. doi:10.1053/j.gastro.2011.04.011. 21621540

[43] Hoarau G, Mukherjee PK, Gower-Rousseau C, Hager C, Chandra J, Retuerto MA, Neut C, Vermeire S, Clemente J, Colombel JF, et al. Bacteriome and Mycobiome Interactions Underscore Microbial Dysbiosis in Familial Crohn’s Disease. mBio. 2016;7(5):e01250-16. 10.1128/mBio.01250-16 PMC5030358 27651359

[44] Derrien M, Collado MC, Ben-Amor K, Salminen S, de Vos WM. The mucin degrader akkermansia muciniphila is an abundant resident of the human intestinal tract. Appl Environ Microbiol. 2008;74(5):1646–1648. doi:10.1128/AEM.01226-07. 18083887 PMC2258631

[45] Glover JS, Ticer TD, Engevik MA. Characterizing the mucin-degrading capacity of the human gut microbiota. Sci Rep. 2022;12(1):8456. doi:10.1038/s41598-022-11819-z. 35589783 PMC9120202

[46] Vaga S, Lee S, Ji B, Andreasson A, Talley NJ, Agréus L, Bidkhori G, Kovatcheva-Datchary P, Park J, Lee D, et al. Compositional and functional differences of the mucosal microbiota along the intestine of healthy individuals. Sci Rep. 2020;10(1):14977. doi:10.1038/s41598-020-71939-2. 32917913 PMC7486370

[47] Gálvez EJC, Iljazovic A, Gronow A, Flavell R, Strowig T. Shaping of intestinal microbiota in Nlrp6- and Rag2-deficient mice depends on community structure. Cell Rep. 2017;21(13):3914–3926. doi:10.1016/j.celrep.2017.12.027. 29281837

[48] Suzuki K, Meek B, Doi Y, Muramatsu M, Chiba T, Honjo T, Fagarasan S. Aberrant expansion of segmented filamentous bacteria in IgA-deficient gut. Proc Natl Acad Sci U S A. 2004;101(7):1981–1986. doi:10.1073/pnas.0307317101. 14766966 PMC357038

[49] Moran JP, Walter J, Tannock GW, Tonkonogy SL, Sartor BR. Bifidobacterium animalis causes extensive duodenitis and mild colonic inflammation in monoassociated interleukin-10-deficient mice. Inflamm Bowel Dis. 2009;15(7):1022–1031. doi:10.1002/ibd.20900. 19235917 PMC2764742

[50] Ward JM, Anver MR, Haines DC, Melhorn JM, Gorelick P, Yan L, Fox JG. Inflammatory large bowel disease in immunodeficient mice naturally infected with Helicobacter hepaticus. Lab Anim Sci. 1996;46:15–20. 8699813

[51] Li X, Fox JG, Whary MT, Yan L, Shames B, Zhao Z. SCID/NCr mice naturally infected with helicobacter hepaticus develop progressive hepatitis, proliferative typhlitis, and colitis. Infect Immun. 1998;66(11):627–638. doi:10.1128/IAI.66.11.5477-5484.1998. 9784560 PMC108686

[52] Buonocore S, Ahern PP, Uhlig HH, Ivanov LDR II, Maloy KJ, Powrie F. Innate lymphoid cells drive interleukin-23-dependent innate intestinal pathology. Nature. 2010;464:1371–1375. 20393462 10.1038/nature08949 PMC3796764

[53] Zhu C, Song K, Shen Z, Quan Y, Tan B, Luo W, Wu S, Tang K, Yang Z, Wang X. Roseburia intestinalis inhibits interleukin‑17 excretion and promotes regulatory T cells differentiation in colitis. Mol Med Rep. 2018;17:7567–7574. 29620246 10.3892/mmr.2018.8833 PMC5983956

[54] Kellermayer R. Roseburia species: prime candidates for microbial therapeutics in inflammatory bowel disease. Gastroenterology. 2019;157:1164–1165. 31356805 10.1053/j.gastro.2019.05.073

[55] Chen L, Wilson JE, Koenigsknecht MJ, Chou WC, Montgomery SA, Truax AD, Brickey WJ, Packey CD, Maharshak N, Matsushima GK, et al. NLRP12 attenuates colon inflammation by maintaining colonic microbial diversity and promoting protective commensal bacterial growth. Nat Immunol. 2017;18:541–551. 28288099 10.1038/ni.3690 PMC5395345

[56] Guo W, Mao B, Cui S, Tang X, Zhang Q, Zhao J, Zhang H. Protective Effects of a Novel Probiotic Bifidobacterium pseudolongum on the Intestinal Barrier of Colitis Mice via Modulating the Pparγ/STAT3 Pathway and Intestinal Microbiota. Foods. 2022;11(1551). 10.3390/foods11111551 PMC9180506 35681301

[57] Pryde SE, Duncan SH, Hold GL, Stewart CS, Flint HJ. The microbiology of butyrate formation in the human colon. FEMS Microbiol Lett. 2002;217:133–139. 12480096 10.1111/j.1574-6968.2002.tb11467.x

[58] Veiga P, Gallini CA, Beal C, Michaud M, Delaney ML, DuBois A, Khlebnikov A, van Hylckama Vlieg JE, Punit S, Glickman JN, et al. Bifidobacterium animalis subsp. lactis fermented milk product reduces inflammation by altering a niche for colitogenic microbes. Proc Natl Acad Sci U S A. 2010;107:18132–18137. 20921388 10.1073/pnas.1011737107 PMC2964251

[59] Lo BC, Shin SB, Canals Hernaez D, Refaeli I, Yu HB, Goebeler V, Cait A, Mohn WW, Vallance BA, McNagny KM. IL-22 preserves gut epithelial integrity and promotes disease remission during chronic. J Immunol. 2019;202:956–965. 30617224 10.4049/jimmunol.1801308

[60] Zitomersky NL, Atkinson BJ, Franklin SW, Mitchell PD, Snapper SB, Comstock LE, Bousvaros A. Characterization of adherent bacteroidales from intestinal biopsies of children and young adults with inflammatory bowel disease. PLoS One. 2013;8:e63686. 23776434 10.1371/journal.pone.0063686 PMC3679120

[61] Kverka M, Zakostelska Z, Klimesova K, Sokol D, Hudcovic T, Hrncir T, Rossmann P, Mrazek J, Kopecny J, Verdu EF, et al. Oral administration of Parabacteroides distasonis antigens attenuates experimental murine colitis through modulation of immunity and microbiota composition. Clin Exp Immunol. 2011;163:250–259. 21087444 10.1111/j.1365-2249.2010.04286.x PMC3043316

[62] Ermann J, Garrett WS, Kuchroo J, Rourida K, Glickman JN, Bleich A, Glimcher LH. Severity of innate immune-mediated colitis is controlled by the cytokine deficiency-induced colitis susceptibility-1 (Cdcs1) locus. Proc Natl Acad Sci U S A. 2011;108:7137–7141. 21482794 10.1073/pnas.1104234108 PMC3084042

[63] Kolodziej LE, Lodolce JP, Chang JE, Schneider JR, Grimm WA, Bartulis SJ, Zhu X, Messer JS, Murphy SF, Reddy N, et al. TNFAIP3 maintains intestinal barrier function and supports epithelial cell tight junctions. PLoS One. 2011;6:e26352. 22031828 10.1371/journal.pone.0026352 PMC3198775

[64] Johansson ME, Hansson GC. Preservation of mucus in histological sections, immunostaining of mucins in fixed tissue, and localization of bacteria with FISH. Methods Mol Biol. 2012;842:229–235. 22259139 10.1007/978-1-61779-513-8_13

[65] Koelink PJ, Wildenberg ME, Stitt LW, Feagan BG, Koldijk M, van T, Wout AB, Atreya R, Vieth M, Brandse JF, et al. Development of reliable, valid and responsive scoring systems for endoscopy and histology in animal models for inflammatory bowel disease. J Crohns Colitis. 2018;12:794–803. 29608662 10.1093/ecco-jcc/jjy035 PMC6022651

[66] Bolyen E, Rideout JR, Dillon MR, Bokulich NA, Abnet CC, Al-Ghalith GA, Alexander H, Alm EJ, Arumugam M, Asnicar F, et al. Reproducible, interactive, scalable and extensible microbiome data science using QIIME 2. Nat Biotechnol. 2019;37:852–857. 31341288 10.1038/s41587-019-0209-9 PMC7015180

[67] Callahan BJ, McMurdie PJ, Rosen MJ, Han AW, Johnson AJ, Holmes SP. DADA2: high-resolution sample inference from Illumina amplicon data. Nat Methods. 2016;13:581–583. 27214047 10.1038/nmeth.3869 PMC4927377

[68] Katoh K, Misawa K, Kuma K, Miyata T. MAFFT: a novel method for rapid multiple sequence alignment based on fast Fourier transform. Nucleic Acids Res. 2002;30:3059–3066. 12136088 10.1093/nar/gkf436 PMC135756

[69] Price MN, Dehal PS, Arkin AP. FastTree: computing large minimum evolution trees with profiles instead of a distance matrix. Mol Biol Evol. 2009;26:1641–1650. 19377059 10.1093/molbev/msp077 PMC2693737

[70] Price MN, Dehal PS, Arkin AP. FastTree 2–approximately maximum-likelihood trees for large alignments. PLoS One. 2010;5:e9490. 20224823 10.1371/journal.pone.0009490 PMC2835736

[71] Faith DP. SYSTEMATICS AND CONSERVATION: ON PREDICTING THE FEATURE DIVERSITY OF SUBSETS OF TAXA. Cladistics. 1992;8:361–373. 34929967 10.1111/j.1096-0031.1992.tb00078.x

[72] Lozupone C, Knight R. UniFrac: a new phylogenetic method for comparing microbial communities. Appl Environ Microbiol. 2005;71:8228–8235. 16332807 10.1128/AEM.71.12.8228-8235.2005 PMC1317376

[73] Lozupone CA, Hamady M, Kelley ST, Knight R. Quantitative and qualitative beta diversity measures lead to different insights into factors that structure microbial communities. Appl Environ Microbiol. 2007;73:1576–1585. 17220268 10.1128/AEM.01996-06 PMC1828774

[74] Bokulich NA, Kaehler BD, Rideout JR, Dillon M, Bolyen E, Knight R, Huttley GA, Gregory Caporaso J. Optimizing taxonomic classification of marker-gene amplicon sequences with QIIME 2ʹs q2-feature-classifier plugin. Microbiome. 2018;6:90. 29773078 10.1186/s40168-018-0470-z PMC5956843

[75] Bokulich NA, Dillon MR, Bolyen E, Kaehler BD, Huttley GA, Caporaso JG. q2-sample-classifier: machine-learning tools for microbiome classification and regression. J Open Res Softw. 2018;3(30). 10.21105/joss.00934 PMC6759219 31552137

[76] McDonald D, Price MN, Goodrich J, Nawrocki EP, DeSantis TZ, Probst A, Andersen GL, Knight R, Hugenholtz P. An improved Greengenes taxonomy with explicit ranks for ecological and evolutionary analyses of bacteria and archaea. ISME J. 2012;6:610–618. 22134646 10.1038/ismej.2011.139 PMC3280142

